# FASN-mediated *de novo* lipogenesis promotes ceramide accumulation and myocardial damage in arrhythmogenic cardiomyopathy

**DOI:** 10.1016/j.isci.2026.117251

**Published:** 2026-08-28

**Authors:** Zirui Liu, Hao Cui, Tianshuo Yang, Songren Shu, Zhice Chen, Jing Han, Mingming Su, Jian Huang, Xiao Chen, Jiangping Song, Shengshou Hu

**Affiliations:** 1 State Key Laboratory of Cardiovascular Disease, Fuwai Hospital, National Center for Cardiovascular Diseases, Chinese Academy of Medical Sciences and Peking Union Medical College, Beijing, China; 2 Department of Cardiovascular Surgery, Fuwai Hospital, National Center for Cardiovascular Diseases, Chinese Academy of Medical Sciences and Peking Union Medical College, Beijing, China; 3 Department of Cardiac Surgery, Fuwai Yunnan Hospital, Chinese Academy of Medical Sciences, Affiliated Cardiovascular Hospital of Kunming Medical University, Kunming, China; 4 Shenzhen Key Laboratory of Cardiovascular Disease, Fuwai Hospital Chinese Academy of Medical Sciences, Shenzhen 518057, China; 5 Beijing Key Laboratory of Xenotransplantation, Animal Experimental Centre, Fuwai Hospital, National Centre for Cardiovascular Disease, Chinese Academy of Medical Sciences and Peking Union Medical College, Beijing 100037, China; 6 Clinical Research Institute, Chinese Academy of Medical Sciences, Beijing 100730, China; 7 Faculty of Clinical Medicine, Peking Union Medical College, Beijing 100730, China

**Keywords:** arrhythmogenic cardiomyopathy, *de novo* lipogenesis, fatty acid synthase, lipotoxicity, myocardial injury

## Abstract

Arrhythmogenic cardiomyopathy (ACM) is a heritable disease caused by desmosomal gene mutations and marked by progressive myocardial loss and fibrofatty replacement. Here, we show that fatty acid synthase (FASN), the rate-limiting enzyme of *de novo* lipogenesis, is upregulated in cardiomyocytes of ACM patients and of a cardiomyocyte-specific Dsg2-mutant mouse model. Desmosomal dysfunction drives nuclear translocation of junction plakoglobin (JUP), which cooperates with SREBP1 to activate FASN transcription. The resulting *de novo* lipogenesis diverts glucose toward synthesis of lipotoxic ceramides, promoting cardiomyocyte apoptosis, as shown by isotope tracing, lipidomics, and RNA sequencing (RNA-seq). Pharmacological FASN inhibition with orlistat reduced myocardial lipid accumulation, apoptosis, inflammation, and fibrosis, improving cardiac function and survival. These findings define a JUP/SREBP1/FASN axis linking desmosomal dysfunction to myocardial lipotoxicity and identify FASN as a candidate therapeutic target in ACM.

## Highlights

• FASN-driven *de novo* lipogenesis is upregulated in ACM cardiomyocytes
• Nuclear JUP cooperates with SREBP1 to drive FASN transcription
• FASN-derived ceramides promote cardiomyocyte apoptosis and myocardial injury
• FASN inhibition with orlistat improves cardiac function and survival in ACM mice

## Graphical abstract

**Figure:**
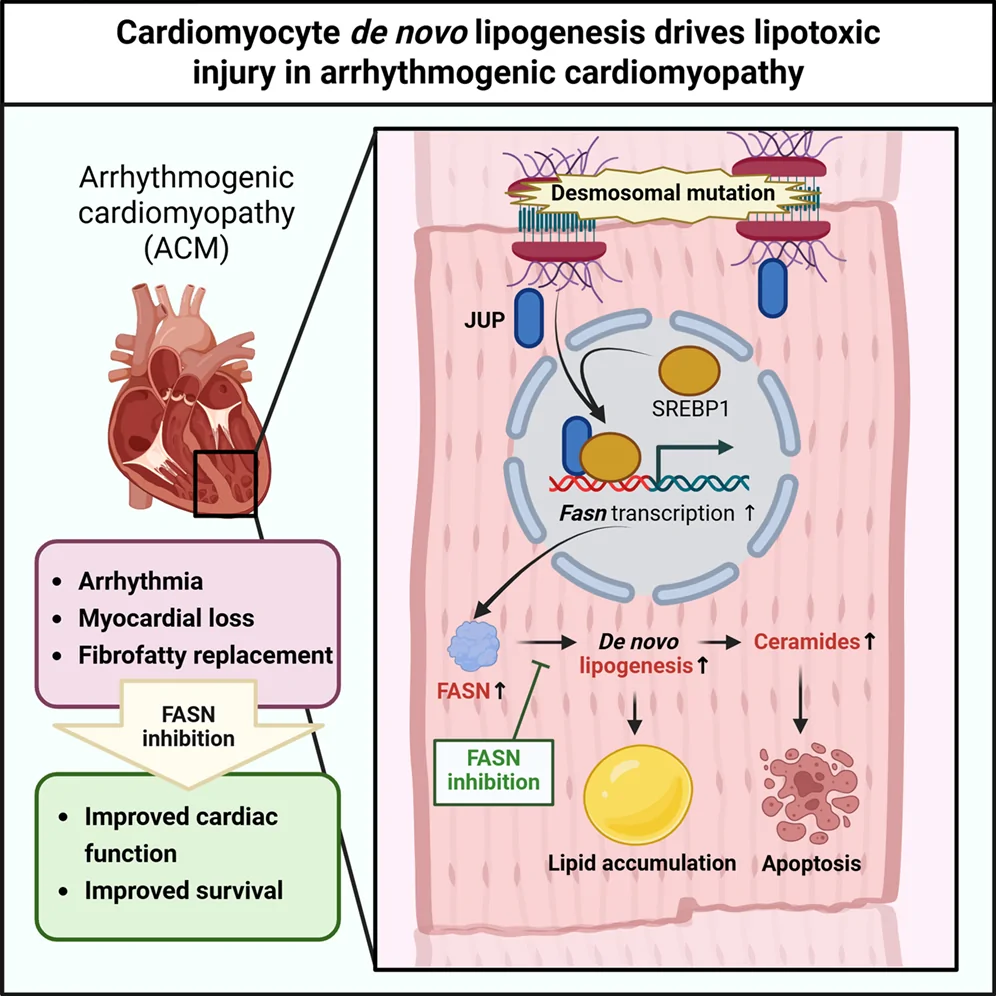

## Teaser

Biological sciences; Molecular biology; Omics; Lipidomics

## Introduction

Arrhythmogenic cardiomyopathy (ACM) represents a familial cardiomyopathy predominantly attributed to mutations in desmosomal genes,^1^ characterized by progressive myocardial loss and replacement by fibrofatty tissue that can involve the right ventricle, left ventricle, or both. The prevalence of ACM is estimated at approximately 1 in 2,000, suggesting a global patient population nearing 3 million individuals.^2^ ACM poses substantial health risks, primarily manifesting as life-threatening ventricular arrhythmias and sudden cardiac death; heart failure occurs in a subset of patients, particularly at advanced stages. Consequently, ACM is a leading cause of sudden cardiac death among young adults, thereby representing a significant global health concern.^3^

Current therapeutic approaches for ACM are limited.^2^ Implantable cardioverter-defibrillators currently represent the sole preventive measure against sudden cardiac death in ACM patients; however, effective pharmacological interventions or treatments to halt the progression of ACM to heart failure are lacking.^1^ Heart transplantation remains the only viable treatment for advanced stages of ACM leading to heart failure. The scarcity of donor organs, however, renders this option inaccessible to the majority of ACM patients. Consequently, the development of pharmacological agents, grounded in the pathological mechanisms of ACM, that can impede or potentially reverse the progression of ACM is imperative for advancing treatment strategies.

The current consensus suggests that the etiology of ACM is associated with mutations in desmosomal genes.^1^^,^^4^ These mutations contribute to ACM’s pathological characteristics, namely the progressive myocardial loss and fibrofatty replacement, with variable biventricular involvement.^5^ A pivotal aspect of ACM research involves elucidating the mechanisms by which desmosomal gene mutations precipitate myocardial cell loss and fibrofatty tissue replacement in the ventricles, along with identifying potential targets for intervention. Notably, Marian and colleagues posited that in ACM, junction plakoglobin (JUP) dissociates from the desmosome and undergoes nuclear translocation, where it competitively suppresses β-catenin/Wnt signaling, thereby promoting adipogenesis.^4^^,^^6^ Previous induced pluripotent stem cell (iPSC)-based modeling studies have demonstrated exaggerated lipogenesis in PKP2-mutant cardiomyocytes via PPAR-γ activation, yet the upstream metabolic enzyme responsible and the specific transcriptional mechanism linking desmosomal dysfunction to lipogenesis remained uncharacterized.^7^ Furthermore, these studies have not adequately explained the predilection for fibrofatty replacement and myocardial loss in the ventricular myocardium, nor have they led to the development of targeted pharmaceutical interventions to reduce myocardial lipid accumulation in ACM. Accumulating evidence indicates that ceramides, a class of sphingolipids, play a causal role in various cardiac pathologies, including heart failure, ischemic heart disease, and atherosclerosis.^8^ Mechanistically, ceramides promote cardiomyocyte apoptosis, induce mitochondrial dysfunction, and drive maladaptive cardiac remodeling.^8^^,^^9^ In healthy hearts, fatty acid synthase (FASN) expression is maintained at very low levels, as *de novo* lipogenesis primarily occurs in the liver and adipose tissue.^10^ Consequently, any marked upregulation of FASN in cardiomyocytes is likely to be pathological.^11^

In this study, we observed *de novo* lipogenesis in cardiomyocytes from both heart samples of ACM patients and a mouse model of ACM induced by point mutations in Dsg2. This process was found to be mediated by FASN. We further discovered that in ACM, mutations in desmosomal genes and the nuclear translocation of JUP, in collaboration with SREBP1, activate and upregulate FASN expression. This *de novo* lipogenesis process leads to the production of lipotoxic compounds, such as ceramides, which contribute to myocardial damage. Our preclinical interventions suggest that FASN may represent a promising therapeutic target for ACM.

## Results

### FASN is upregulated in cardiomyocytes of human ACM hearts

**Figure 1:**
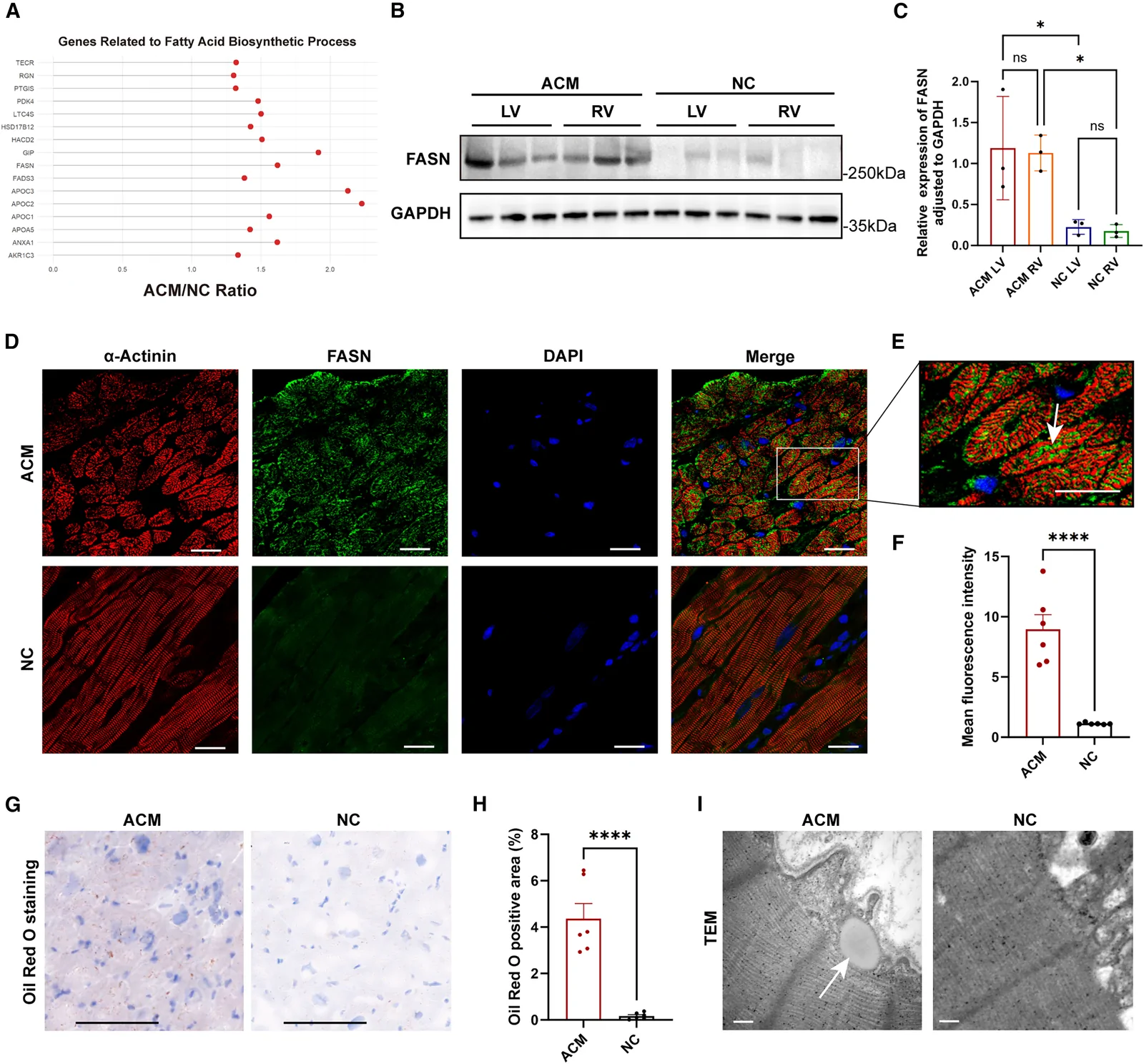
FASN is upregulated in cardiomyocytes of ACM patients (A) TMT-based proteomics heatmap showing upregulation of fatty acid synthesis pathway proteins in ACM (*n* = 4 per group) versus non-diseased controls (NC). Data are log_2_-transformed and *Z* scored. (B) Representative western blots of FASN and GAPDH in left ventricular (LV) and right ventricular (RV) myocardium from ACM patients and NC (*n* = 3 per group). (C) Densitometric quantification of FASN normalized to GAPDH from (B). Each symbol represents an individual patient (*n* = 3 per group). Data are mean ± SEM. ∗*p* < 0.05; ns, not significant (two-way ANOVA with Tukey’s post hoc test). (D) Immunofluorescence staining of human heart sections for α-actinin (red), FASN (green), and DAPI (blue). The white box indicates the region shown at higher magnification in (E). Scale bars, 25 μm. (E) Higher-magnification view of the boxed area in (D), arrow showing FASN (green) localized in the cytoplasm of α-actinin-positive cardiomyocytes (red). Scale bars, 25 μm. (F) Quantification of FASN fluorescence intensity in α-actinin-positive cardiomyocytes (*n* = 6 patients per group). ∗∗∗∗*p* < 0.0001 (Student’s *t* test). (G) Oil red O staining of human ACM and NC myocardium. Lipid droplets are stained red. Scale bars, 50 μm. (H) Quantification of oil red O-positive area as a percentage of total myocardial area (*n* = 6 per group). ∗∗∗∗*p* < 0.0001 (Student’s *t* test). (I) Transmission electron microscopy of ACM and NC myocardium. Arrows indicate sub-sarcolemmal lipid droplets. Scale bars, 500 nm.

To investigate whether *de novo* lipogenesis occurs in the hearts of ACM patients, we performed tandem mass tag (TMT) labeled proteomic analysis on human ACM and non-diseased control (NC) myocardial tissues. Among lipid metabolism-related proteins, FASN, the rate-limiting enzyme of *de novo* lipogenesis, was significantly upregulated in ACM samples (Figure 1A). Western blot analysis of carefully dissected left ventricular (LV) and right ventricular (RV) myocardium and adipose tissue showed that FASN upregulation was confined to the myocardial compartment, with no significant change in adipose tissue (Figures 1B, 1C, and S1A–S1C). Notably, FASN levels were elevated in both LV and RV myocardium of ACM patients compared to NC. This upregulation appeared specific to ACM, as no difference in FASN expression was observed between dilated cardiomyopathy (DCM) and NC hearts (Figures S1D and S1E).

To determine which cell types express FASN in ACM hearts, we performed immunofluorescence staining for FASN together with the cardiomyocyte marker α-actinin. Confocal imaging revealed that FASN signal was predominantly localized in α-actinin-positive cardiomyocytes, where it appeared diffusely distributed in the cytoplasm rather than aligned with the myofibrils (Figures 1D–1F).

Consistent with increased FASN expression, oil red O staining showed abundant lipid droplet accumulation within the myocardium of ACM hearts, whereas NC hearts displayed little or no staining (Figures 1G and 1H). Transmission electron microscopy further revealed frequent sub-sarcolemmal lipid droplets in ACM cardiomyocytes, which were absent in NC controls (Figure 1I). Together, these data indicate that FASN is specifically upregulated in ACM cardiomyocytes and is associated with intracellular lipid accumulation.

### FASN is upregulated in the myocardium of Dsg2^mut/mut^ mice

**Figure 2:**
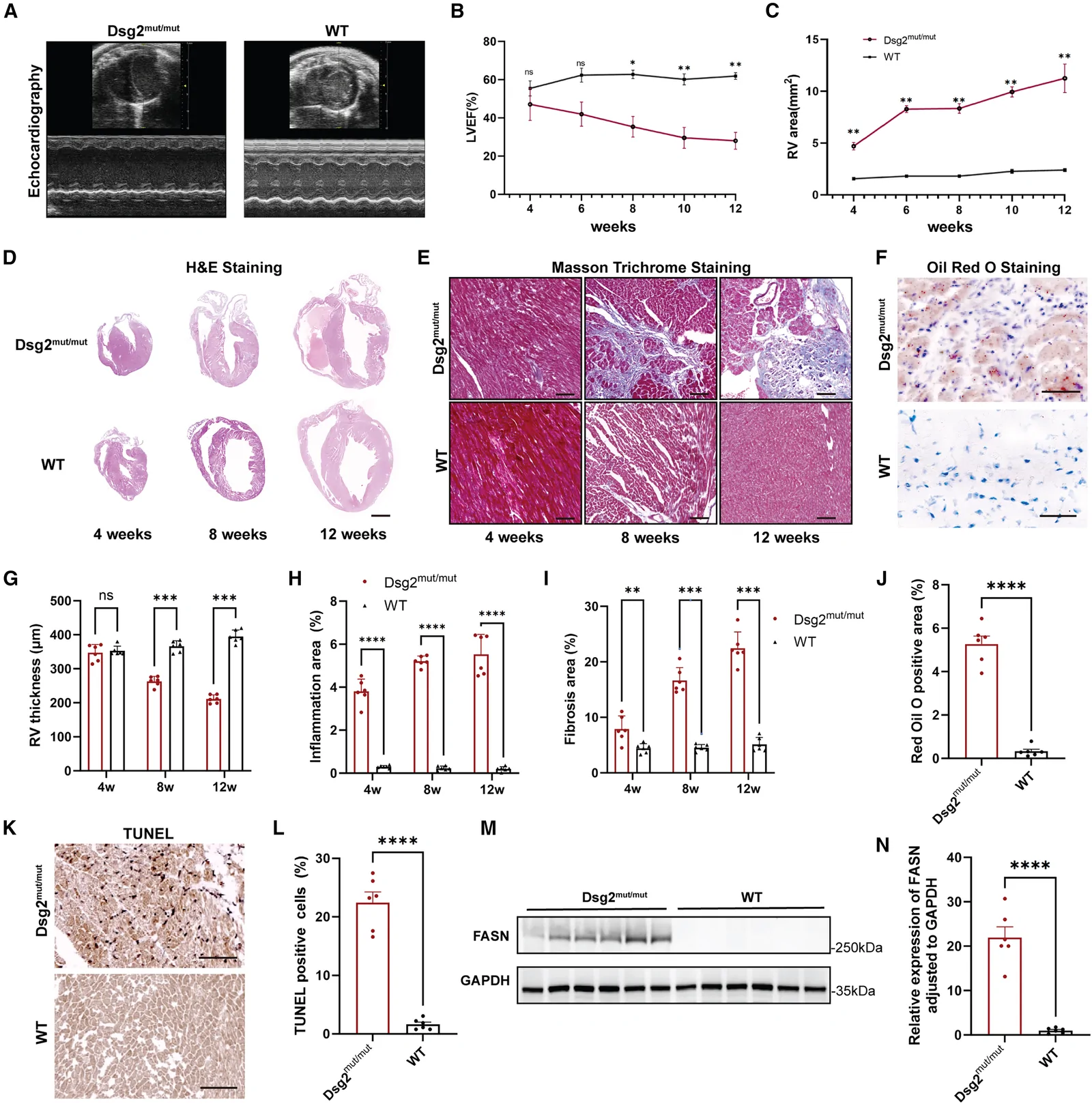
The constructed Dsg2^mut/mut^ transgenic mice exhibit upregulation of FASN (A) Representative B-mode and M-mode echocardiographic images of the left ventricle in WT and Dsg2^mut/mut^ mice at 12 weeks of age. (B) Longitudinal analysis of left ventricular ejection fraction (LVEF, %) from 4 to 12 weeks of age (*n* = 8 per genotype). ∗*p* < 0.05, ∗∗*p* < 0.01 vs. WT (two-way ANOVA with Holm-Šídák test). (C) Right ventricular area (RV area, mm^2^) over time (*n* = 8 per genotype). ∗∗*p* < 0.01 vs. WT. (D) Whole-heart H&E staining at 4, 8, and 12 weeks. Scale bars, 1 mm. (E) Masson’s trichrome staining. Fibrosis is shown in blue. Scale bars, 100 μm. (F) Oil red O staining of mouse hearts at 12 weeks. Scale bars, 50 μm. (G) Quantification of RV thickness (μm) from H&E sections (*n* = 6 per group). ∗∗∗*p* < 0.001 vs. WT (Student’s *t* test). (H) Quantification of inflammation infiltration area (%) from H&E staining (*n* = 6 per group). ∗∗∗∗*p* < 0.0001 vs. WT (Student’s *t* test). (I) Quantification of fibrotic area (%) from Masson’s staining (*n* = 6 per group). ∗∗*p* < 0.01, ∗∗∗*p* < 0.001 vs. WT (Student’s *t* test). (J) Quantification of oil red O positive area (%) (*n* = 6 per group). ∗∗∗∗*p* < 0.0001 vs. WT (Student’s *t* test). (K) TUNEL staining of mouse hearts at 12 weeks. Scale bars, 200 μm. (L) Quantification of TUNEL positive cells (%) (*n* = 6 per group). ∗∗∗∗*p* < 0.0001 vs. WT (Student’s *t* test). (M) Western blot of FASN and GAPDH in whole-heart lysates at 12 weeks (*n* = 6 per genotype). (N) Densitometric quantification of FASN normalized to GAPDH from (M). ∗∗∗∗*p* < 0.0001 (Student’s *t* test).

To investigate the functional role of FASN upregulation in ACM, we generated a knockin mouse model carrying the Dsg2 p.F536C mutation (Figures S2A and S2B). Homozygous Dsg2^mut/mut^ mice developed a progressive cardiac phenotype. Echocardiography revealed a progressive decline in LV ejection fraction (LVEF) from 8 weeks onward, reaching heart failure levels by 12 weeks (Figures 2A and 2B). RV area was also significantly increased starting at 4 weeks (Figure 2C). Starting from 4 weeks of age, they exhibited inflammatory infiltration and fibrosis, which worsened over time (Figures 2D, 2E, and 2H–2I). Both ventricles showed pathological alterations, although the right ventricle displayed a more pronounced decrease in wall thickness (Figure 2G) and lipid accumulation (Figures 2F and 2J). TUNEL staining further revealed a marked increase in apoptotic cardiomyocytes in mutant hearts compared with wild-type (WT) littermates (Figures 2K and 2L). Consistent with our findings in ACM patients, western blotting demonstrated that FASN protein levels were markedly higher in Dsg2^mut/mut^ hearts compared to WT littermates at 12 weeks (Figures 2M and 2N). Together, these data suggest that Dsg2 mutation leads to FASN upregulation, myocardial lipid accumulation, inflammation, and fibrosis, recapitulating key features of human ACM.

### Nuclear translocation of JUP cooperates with SREBP1 to activate FASN transcription in ACM

**Figure 3:**
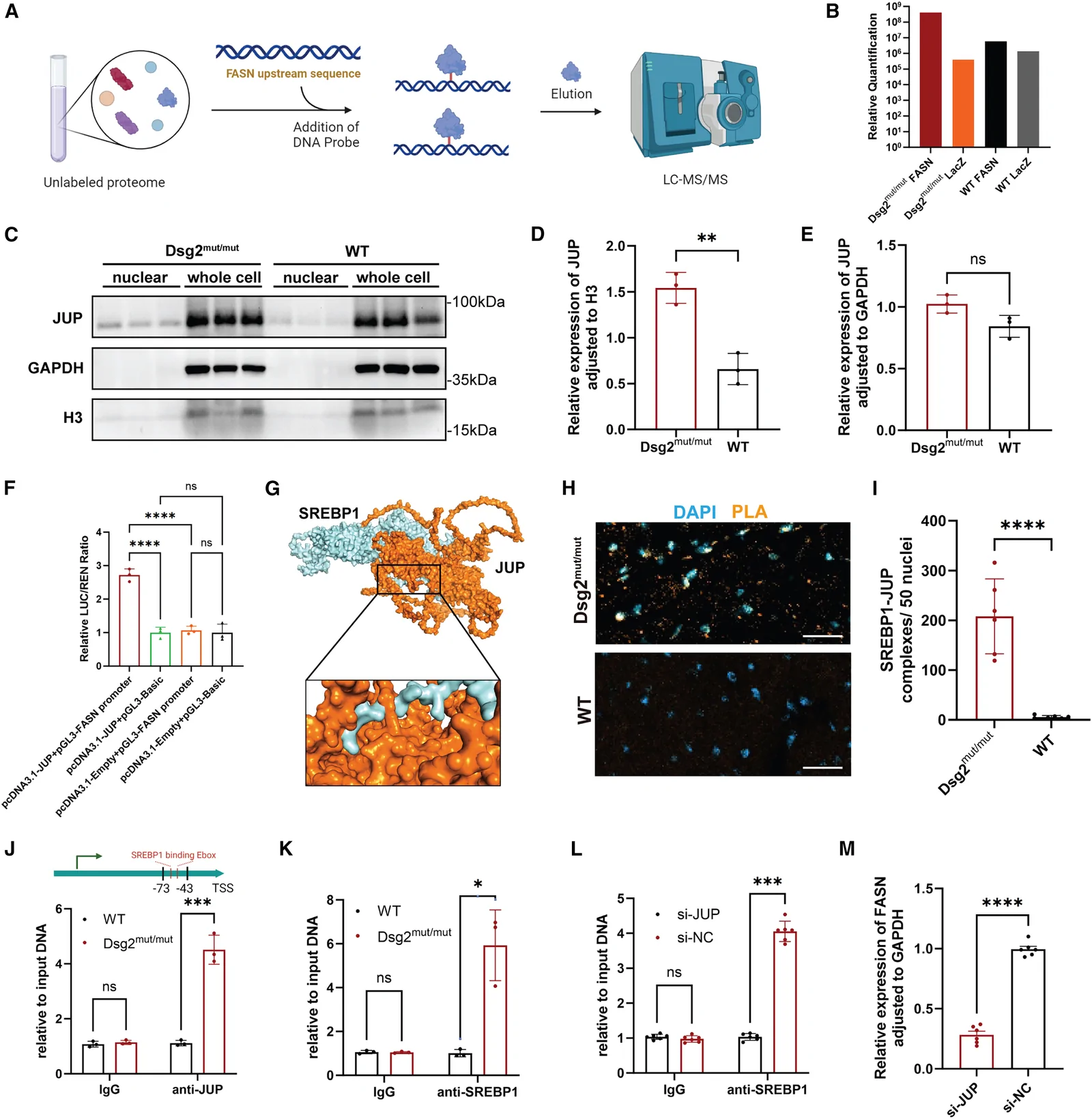
JUP synergizes with SREBP1 in the nucleus to activate FASN transcription (A) Schematic of DNA pull-down using biotinylated FASN promoter probes followed by liquid chromatography-mass spectrometry (LC-MS)/MS. (B) MS quantification of JUP binding to FASN probe vs. LacZ control in WT and Dsg2^mut/mut^ hearts. Data are log_10_ relative quantification (*n* = 3). (C) Nuclear-cytoplasmic fractionation western blots of JUP, GAPDH, and Histone H3 in WT and Dsg2^mut/mut^ hearts (*n* = 3). (D) Quantification of JUP in the nuclear fraction (normalized to H3). ∗∗*p* < 0.01 (Student’s *t* test). (E) Quantification of JUP in whole-cell lysate (normalized to GAPDH). (Student’s *t* test). (F) Dual-luciferase reporter assay in HL-1 cells co-transfected with JUP expression vector or empty vector and FASN promoter luciferase construct or basic vector. Relative luciferase activity (firefly/Renilla) is shown (*n* = 3). ∗∗∗∗*p* < 0.0001; ns, not significant (one-way ANOVA with Tukey’s test). (G) Predicted molecular docking of JUP and SREBP1 (surface representation). (H) Duolink PLA (proximity ligation assay) in WT and Dsg2^mut/mut^ hearts showing JUP-SREBP1 proximity (orange dots). Scale bars, 50 μm. (I) Quantification of PLA signals per 50 nuclei (*n* = 6). ∗∗∗∗*p* < 0.0001 (Student’s *t* test). (J) ChIP-qPCR using anti-JUP antibody on chromatin from WT and Dsg2^mut/mut^ hearts. qPCR primers target the SREBP1 binding motif (−73 to −42 bp) in the FASN promoter. Data are % input (mean ± SEM, *n* = 3). ∗∗∗*p* < 0.001 vs. WT IgG; ns, not significant (two-way ANOVA). (K) ChIP-qPCR using anti-SREBP1 antibody on the same samples. ∗*p* < 0.05 vs. WT (two-way ANOVA). (L) ChIP-qPCR for SREBP1 in sh-Dsg2 HL-1 cells transfected with control siRNA (siNC) or JUP siRNA (siJUP). JUP knockdown reduces SREBP1 occupancy. Data are % input (*n* = 6). ∗∗∗*p* < 0.001 (Student’s *t* test). (M) FASN mRNA expression in sh-Dsg2 HL-1 cells after siNC or siJUP, measured by qPCR (*n* = 6). ∗∗∗∗*p* < 0.0001 (Student’s *t* test).

FASN expression is mainly regulated at the transcriptional level. We conducted qPCR experiments and found that the transcripts of FASN were significantly upregulated in both human and Dsg2^mut/mut^ mouse hearts (Figures S3A and S3B), suggesting transcriptional activation. To identify upstream regulators, we designed DNA probes for 2,000 bp upstream of the FASN promoter, followed by mass spectrometry proteomics (Figure 3A). The DNA pull-down results showed that JUP binds to the FASN promoter in ACM hearts (Figure 3B), which was further validated by western blot of the pull-down material (Figure S3D). Nuclear-cytoplasmic fractionation and immunofluorescence showed increased JUP nuclear translocation in Dsg2^mut/mut^ hearts (Figures 3C–3E and S3C). A dual-luciferase reporter assay confirmed that JUP overexpression in HL-1 cells enhances FASN promoter activity (Figures 3F and S3E).

Since JUP is not a canonical transcription factor, we explored whether it cooperates with known regulators of FASN transcription, including SREBP1, ChREBP, LXRα, and USF-1.^10^^,^^12^ Protein interaction predictions indicated that JUP binds most strongly to SREBP1, with the interaction predicted to involve the N-terminal nuclear entry region of SREBP1 (Figure 3G). Duolink proximity ligation assays (PLAs) showed a marked increase in JUP-SREBP1 proximity in Dsg2^mut/mut^ hearts compared to WT (Figures 3H and 3I). To determine whether JUP and SREBP1 co-occupy the FASN promoter, we performed chromatin immunoprecipitation (ChIP)-qPCR. Using an anti-JUP antibody, we observed significantly higher JUP occupancy at the SREBP1 binding motif (−73 to −42 bp) of the FASN promoter in Dsg2^mut/mut^ mice (Figure 3J). Using an anti-SREBP1 antibody, we found that SREBP1 binding to the same region was also increased in Dsg2^mut/mut^ hearts (Figure 3K). To further dissect the molecular mechanism, we established a Dsg2-knockdown HL-1 cell line (sh-Dsg2) using lentiviral short hairpin RNA (shRNA), which achieved approximately 90% reduction in Dsg2 expression (Figures S3F and S3G). Importantly, knockdown of JUP in Dsg2-deficient HL-1 cells reduced SREBP1 occupancy (Figure 3L) and decreased FASN mRNA expression (Figure 3M), indicating that JUP contributes to efficient SREBP1 recruitment and FASN transactivation. Together, these findings suggest that desmosomal dysfunction promotes JUP nuclear translocation, where JUP facilitates SREBP1 binding to the FASN promoter, thereby driving *de novo* lipogenesis in ACM.

### FASN facilitates *de novo* lipogenesis in ACM

**Figure 4:**
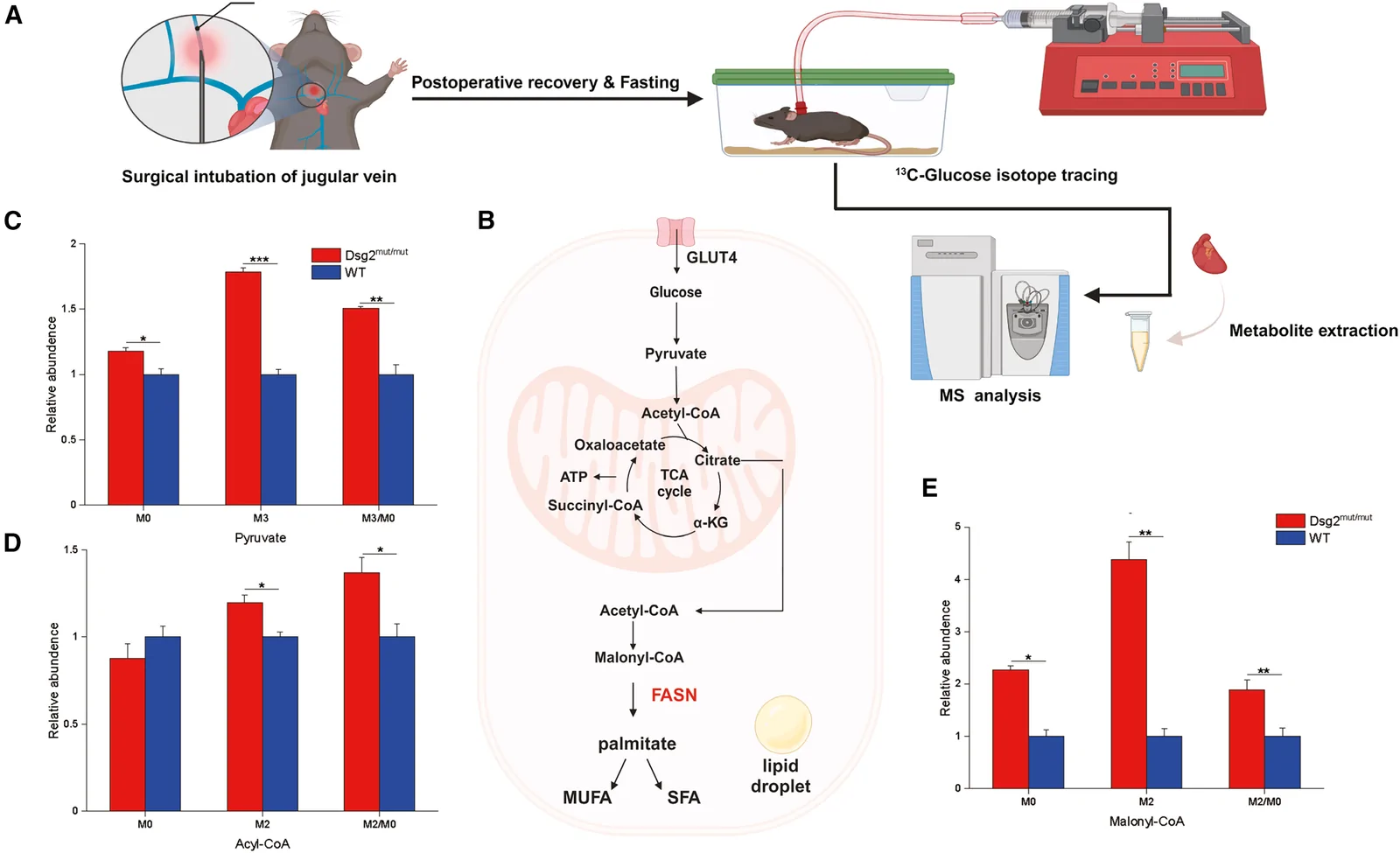
Isotope tracing indicates *de novo* lipogenesis in Dsg2^mut/mut^ mouse hearts (A) Schematic representation of *in vivo*^13^C-glucose infusion via jugular vein catheterization. (B) Simplified metabolic pathway illustrating the conversion of glucose to pyruvate, acyl-CoA, malonyl-CoA, and downstream fatty acids. (C–E) Mass spectrometry quantification of ^13^C-labeled (red) and unlabeled (blue) pyruvate (C), acyl-CoA (D), and malonyl-CoA (E) in hearts of wild-type (WT) and Dsg2^mut/mut^ mice (*n* = 5 per genotype). Data are mean ± SEM. ∗*p* < 0.05, ∗∗*p* < 0.01, ∗∗∗*p* < 0.001 (Student’s *t* test).

FASN plays a pivotal role in mediating *de novo* fatty acid synthesis by converting substrates, such as glucose, into palmitic acid, which is subsequently used to form other fatty acids. To investigate whether the upregulation of FASN in ACM can drive *de novo* lipogenesis *in vivo*, we employed a ^13^C-glucose isotope tracing approach (Figure 4A). Key intermediates of the lipogenic cascade, including pyruvate, acyl-CoA, and malonyl-CoA (Figure 4B), were analyzed as crucial substrates in this biosynthetic pathway. After isotope tracing, metabolites were extracted from the hearts of WT and Dsg2^mut/mut^ mice, and the abundance and isotopic enrichment of these metabolites were measured by mass spectrometry (Figures 4C–4E). Compared to WT, Dsg2^mut/mut^ hearts showed significantly higher levels of ^13^C-labeled pyruvate, acyl-CoA, and malonyl-CoA, indicating increased flux through the *de novo* lipogenesis pathway. These data suggest that FASN upregulation in ACM promotes the conversion of glucose into fatty acid precursors in the myocardium.

### *De novo* lipogenesis-induced ceramides mediate myocardial injury

**Figure 5:**
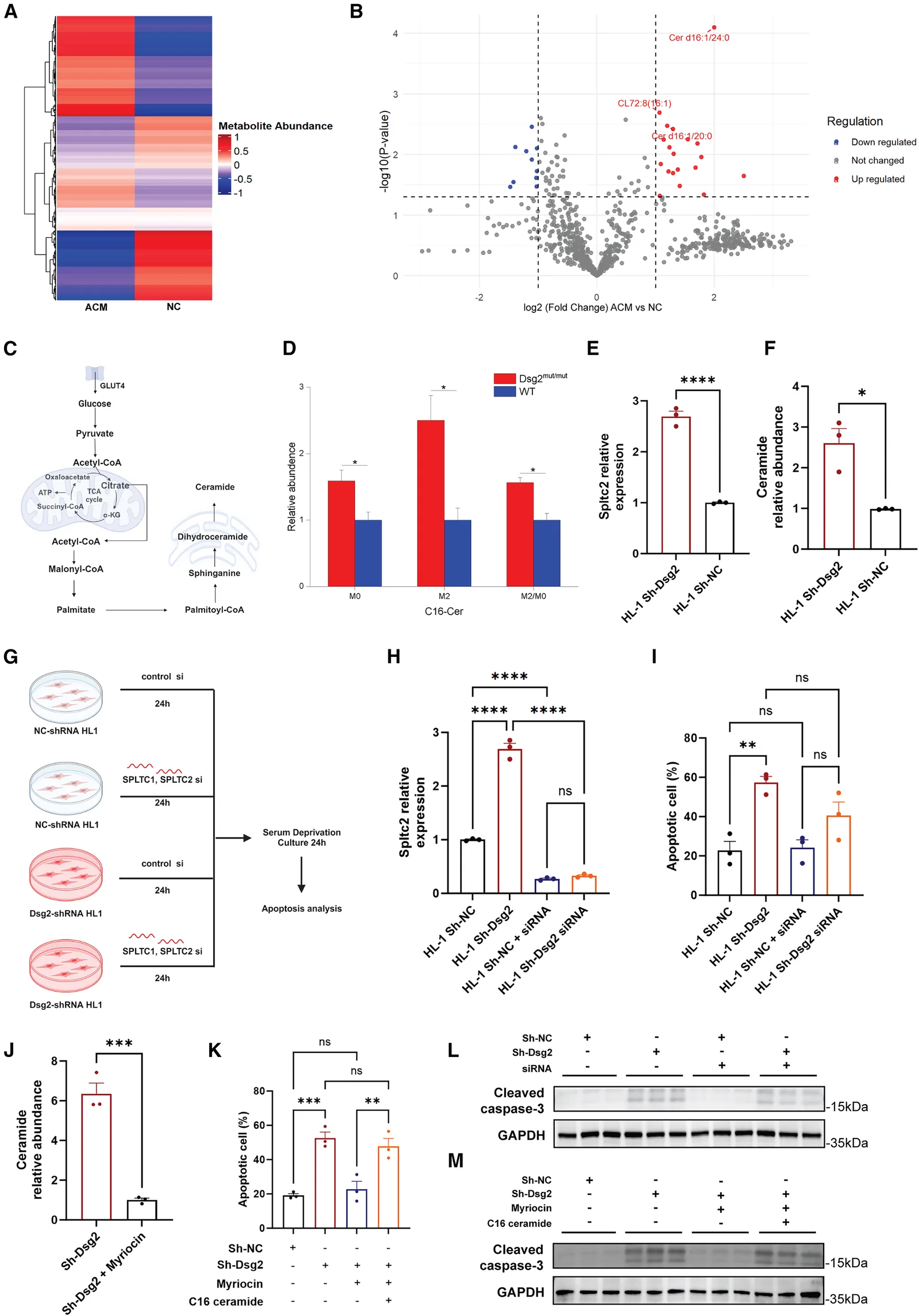
Excess ceramides trigger cardiomyocyte death after *de novo* synthesis of lipids (A) Heatmap of lipidomics data from human ACM and non-diseased control (NC) myocardium (*n* = 5 per group). Data are log_2_-transformed and *Z* scored. (B) Volcano plot of lipid species; upregulated lipids are highlighted in red. (C) Simplified pathway diagram illustrating the conversion of glucose into ceramide. (D) ^13^C-glucose isotope tracing: relative abundance of ^13^C-labeled ceramide in Dsg2^mut/mut^ vs. wild-type (WT) hearts (*n* = 5). ∗*p* < 0.05 (Student’s *t* test). (E) qPCR of Sptlc2 in HL-1 sh-NC and sh-Dsg2 cells (*n* = 3). ∗∗∗∗*p* < 0.0001 (Student’s *t* test). (F) Relative ceramide abundance in sh-NC vs. sh-Dsg2 cells measured by LC-MS/MS (*n* = 3). ∗*p* < 0.05 (Student’s *t* test). (G) Experimental design for serum deprivation and siRNA targeting SPTLC1/2. (H) qPCR validation of Sptlc2 knockdown efficiency in indicated groups (*n* = 3). ∗∗∗∗*p* < 0.0001 (one-way ANOVA with Tukey’s test). (I) Apoptosis quantified by annexin V/propidium iodide (PI) flow cytometry in the groups shown in (G) (*n* = 3). ∗∗*p* < 0.01; ns, not significant (one-way ANOVA with Tukey’s test). (J) Ceramide relative abundance in sh-Dsg2 cells treated with vehicle vs. myriocin measured by LC-MS/MS (*n* = 3). ∗∗∗*p* < 0.001 (Student’s *t* test). (K) Annexin V/PI apoptosis analysis in sh-NC, sh-Dsg2, sh-Dsg2 + myriocin, and sh-Dsg2 + myriocin + C16-ceramide (*n* = 4). ∗∗*p* < 0.01, ∗∗∗*p* < 0.001 (one-way ANOVA with Tukey’s test). (L) Western blot of cleaved caspase-3 and GAPDH under the same conditions as (I). (M) Western blot of cleaved caspase-3 and GAPDH for the groups in (K). Data are mean ± SEM. ∗*p* < 0.05, ∗∗*p* < 0.01, ∗∗∗*p* < 0.001, ∗∗∗∗*p* < 0.0001; ns, not significant.

Lipidomic analysis of ACM myocardial tissues revealed substantial differences compared to non-diseased controls (NC), with the most pronounced changes observed in ceramide species (Figures 5A, 5B, and S4). To determine whether these ceramides originate from *de novo* synthesized fatty acids, we performed ^13^C-glucose isotope tracing in Dsg2^mut/mut^ mice. Both total ceramide levels and the incorporation of ^13^C-glucose into ceramide were significantly increased in Dsg2^mut/mut^ hearts compared to WT (Figures 5C and 5D). RNA sequencing (RNA-seq) and Gene Ontology (GO) enrichment analysis showed upregulation of genes involved in ceramide synthesis and lipid transport in Dsg2^mut/mut^ hearts (Figure S5), suggesting that transcriptional activation contributes to ceramide accumulation.

To directly examine the role of ceramides in cardiomyocyte injury, we used the sh-Dsg2 HL-1 cell line (Figures S3F and S3G). These cells exhibited upregulation of the ceramide synthase Sptlc2 (Figure 5E) and elevated ceramide levels (Figure 5F). Under serum deprivation, sh-Dsg2 cells showed increased apoptosis, which was significantly reduced by small interfering RNA (siRNA)-mediated knockdown of Sptlc1/2 (Figures 5G–5I). To further establish causality, we treated sh-Dsg2 cells with myriocin, a pharmacological inhibitor of ceramide synthesis. Myriocin reduced both ceramide levels and apoptosis, and this effect was partially reversed by adding exogenous C16-ceramide (Figures 5J and 5K). Consistently, cleaved caspase-3 levels followed the same pattern (Figures 5L and 5M). These results suggest that ceramide production downstream of FASN-mediated *de novo* lipogenesis directly contributes to cardiomyocyte apoptosis.

To explore the clinical relevance of ceramide accumulation in ACM, we performed an exploratory analysis of plasma samples from a cohort of ACM patients and healthy relatives (Table S3). Lipidomics revealed that ACM patients who had experienced major adverse cardiovascular events (MACE) exhibited significantly higher plasma ceramide concentrations compared to both ACM patients without MACE and healthy relatives (Figure S6A). A forest plot from univariable analysis suggested that plasma ceramide levels were associated with MACE (Figure S6B).

### FASN is a potential therapeutic target for ACM

**Figure 6:**
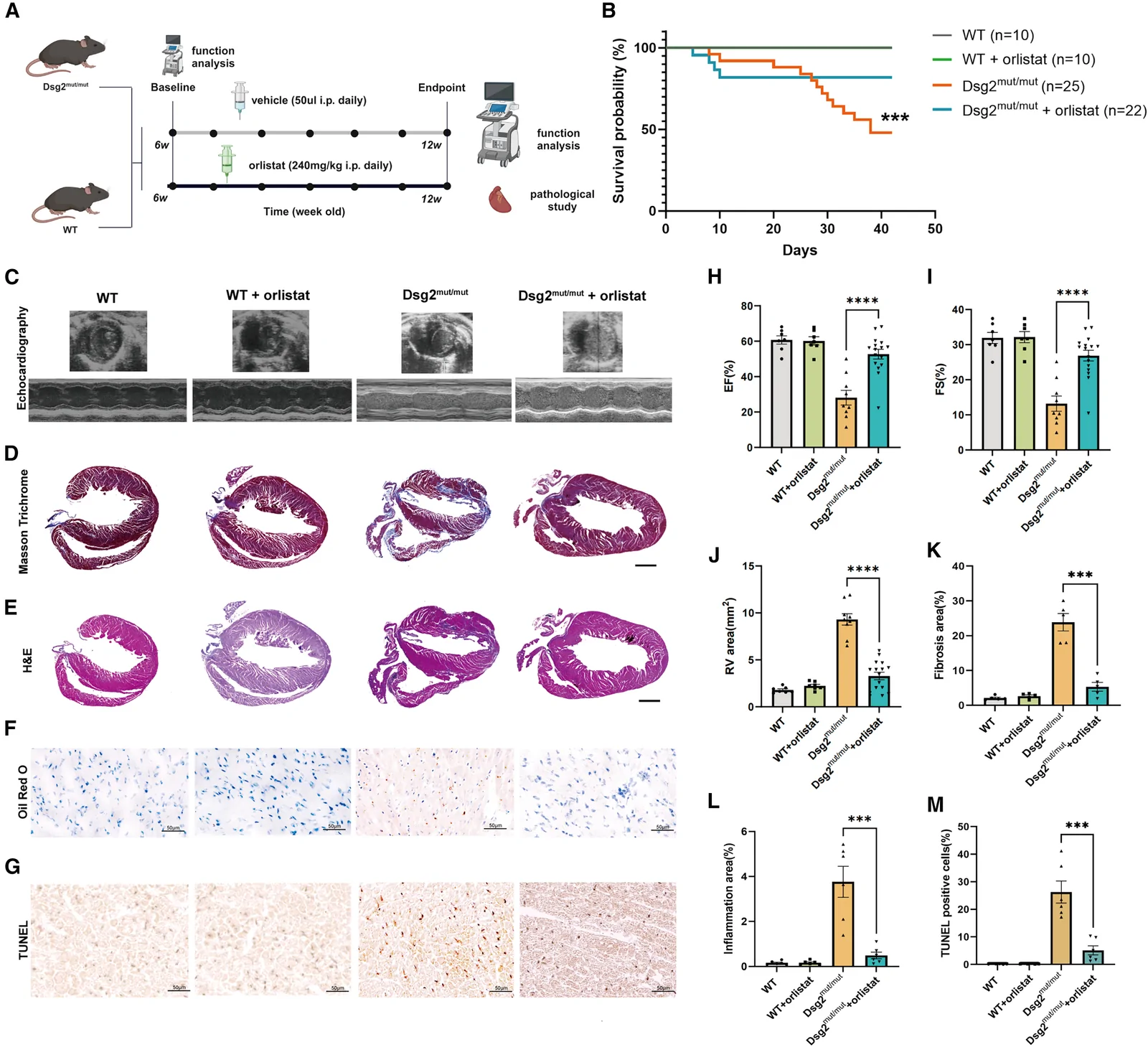
FASN is a potential therapeutic target for ACM (A) Experimental timeline: 6-week-old Dsg2^mut/mut^ mice and littermate wild-type (WT) controls received daily intraperitoneal injections of the FASN inhibitor orlistat or vehicle for 6 weeks. (B) Kaplan-Meier survival curves (*n* = 10 WT + vehicle, 10 WT + orlistat, 25 Dsg2^mut/mut^ + vehicle, and 22 Dsg2^mut/mut^ + orlistat). ∗∗*p* < 0.01 (log rank test). (C) Representative B-mode and M-mode echocardiographic images of the left ventricle at 12 weeks. (D) Masson’s trichrome staining of heart sections. Scale bars, 1 mm. (E) Hematoxylin and eosin (H&E) staining of heart sections. Scale bars, 1 mm. (F) Oil red O staining of heart sections. Scale bars, 50 μm. (G) TUNEL staining of heart sections. Scale bars, 50 μm. (H–J) Quantification of left ventricular ejection fraction (LVEF, %) (H), fractional shortening (FS, %) (I), and right ventricular area (RV area, mm^2^) (J) (*n* = 15 per group). ∗∗∗∗*p* < 0.0001 (two-way ANOVA with Holm-Šídák test). (K) Quantification of fibrotic area (%) from Masson’s trichrome staining (*n* = 6 per group). ∗∗∗*p* < 0.001 (two-way ANOVA with Holm-Šídák test). (L) Quantification of inflammatory infiltration area (%) from H&E staining (*n* = 6 per group). ∗∗∗*p* < 0.001 (two-way ANOVA with Holm-Šídák test). (M) Quantification of TUNEL-positive cells per field (*n* = 6 per group). ∗∗∗*p* < 0.001 (two-way ANOVA with Holm-Šídák test). Data are mean ± SEM. WT + vehicle and WT + orlistat groups were indistinguishable in all parameters; only comparisons between Dsg2^mut/mut^ groups are shown.

To investigate whether FASN could serve as a therapeutic target in ACM, we treated 6-week-old Dsg2^mut/mut^ mice with the clinically established FASN inhibitor orlistat or vehicle control for 6 weeks (Figure 6A). Orlistat treatment significantly improved the survival of Dsg2^mut/mut^ mice compared to vehicle (Figure 6B). Echocardiographic assessment revealed that orlistat prevented the progressive decline in LVEF and fractional shortening (FS) observed in vehicle-treated mutants, and also reduced RV dilation (Figures 6C and 6H–6J). These functional improvements were accompanied by a striking reduction in myocardial lipid droplet accumulation (Figure 6F), fewer apoptotic cells (Figures 6G and 6M), and diminished inflammatory infiltration (Figures 6E and 6L) in orlistat-treated Dsg2^mut/mut^ hearts. Masson’s trichrome staining demonstrated a significant decrease in myocardial fibrosis (Figures 6D and 6K). Collectively, these data suggest that pharmacological inhibition of FASN attenuates key pathological features of ACM, thereby preserving cardiac function.

**Figure 7:**
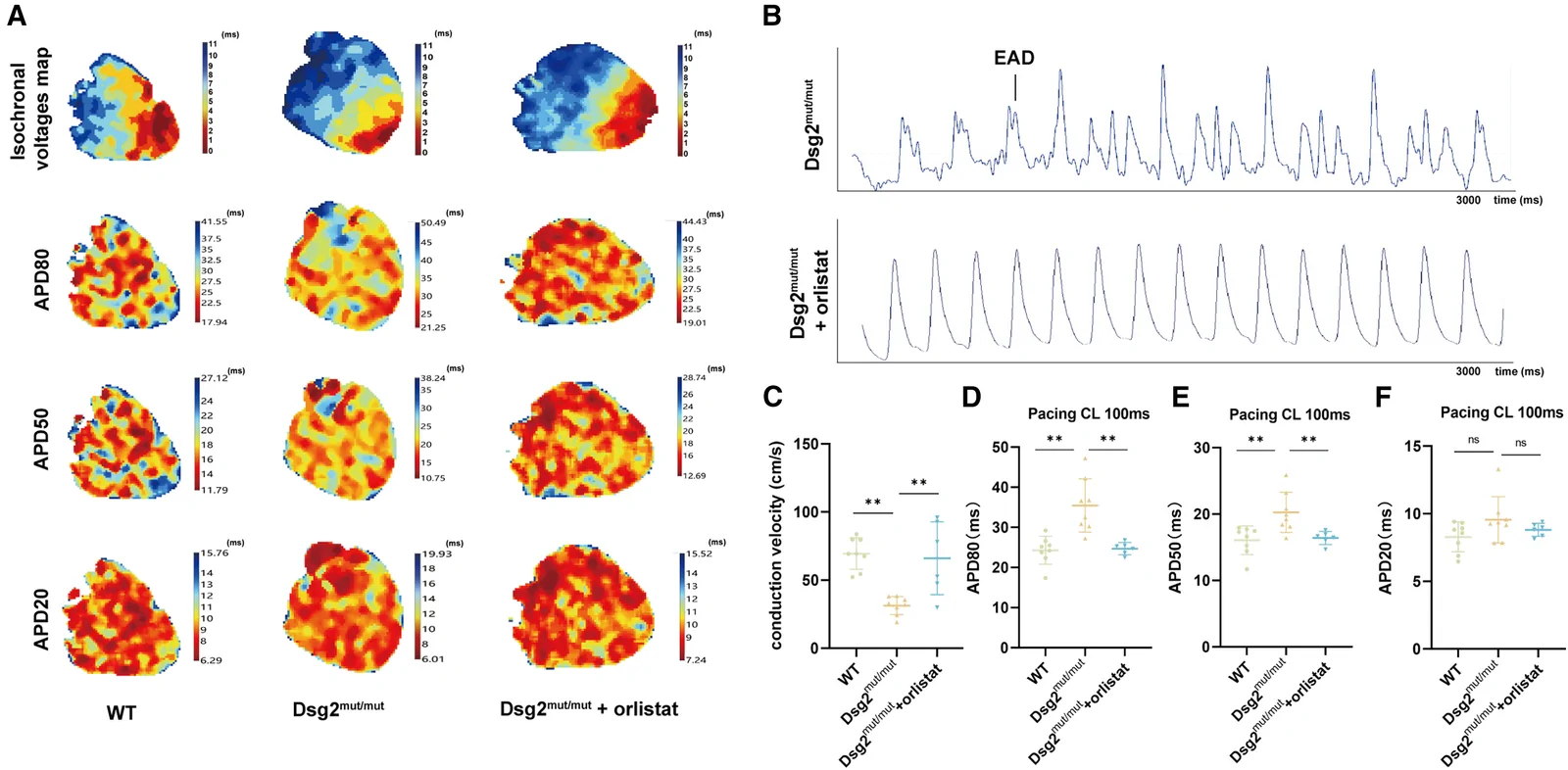
Pharmacological FASN inhibition ameliorates electrophysiological abnormalities of ACM hearts (A) Representative isochronal activation maps and action potential duration (APD) maps at a pacing cycle length of 100 ms in hearts from WT + vehicle, Dsg2^mut/mut^ + vehicle, and Dsg2^mut/mut^ + orlistat groups. (B) Representative surface electrograms (ECGs) of Dsg2^mut/mut^ + vehicle (upper image) and Dsg2^mut/mut^ + orlistat (lower image). Arrowheads indicate early afterdepolarizations (EADs). (C) Quantification of conduction velocity at a 100-ms pacing cycle length (*n* = 6 per group). ∗∗*p* < 0.01 (one-way ANOVA with Tukey’s multiple-comparisons test). (D–F) Quantification of action potential duration at 80% (APD80, D), 50% (APD50, E), and 20% (APD20, F) repolarization at a pacing cycle length of 160 ms (*n* = 6 per group). ∗*p* < 0.05, ∗∗*p* < 0.01 vs. Dsg2^mut/mut^ + vehicle (one-way ANOVA with Tukey’s multiple-comparisons test). Data are mean ± SEM.

Given that life-threatening arrhythmias are a major clinical manifestation of ACM, we next examined whether orlistat treatment improves cardiac electrophysiology. *Ex vivo* optical mapping was performed on hearts isolated from mice after the 6-week treatment regimen (Figure 6A). During sinus rhythm, Dsg2^mut/mut^ hearts frequently exhibited early afterdepolarizations (EADs), which are considered precursors of spontaneous ventricular tachycardia (Figure 7B, upper). Orlistat treatment markedly reduced the incidence of EADs (Figure 7B, lower). Isochronal activation mapping and activation duration mapping were used to quantify action potential duration (APD) and conduction velocity (Figure 7A). Compared to vehicle, orlistat-treated Dsg2^mut/mut^ hearts showed significantly faster conduction velocity at a pacing cycle length of 100 ms (Figure 7C). In addition, orlistat shortened APD at 80% and 50% repolarization, but not at 20% repolarization (Figures 7D–7F). The enhanced conduction velocity and shortened APD are consistent with reduced arrhythmic risk. These findings suggest that FASN inhibition not only halts structural deterioration but also improves electrical stability in ACM mice. Together, our results support the concept that targeting FASN represents a promising therapeutic strategy for ACM.

## Discussion

ACM is a highly complex myocardial disease caused by mutations in the desmosomal genes. Its pathological hallmarks include desmosomal gene mutations, fibrofatty replacement, myocardial loss, and susceptibility to lethal arrhythmias.^1^^,^^3^ Previous studies have focused on the origin of adipocytes in ACM, proposing mechanisms, such as transdifferentiation of cardiomyocytes or differentiation of epicardial progenitors.^6^^,^^13^^,^^14^^,^^15^ While these hypotheses provide insights into the source of adipose tissue, they have not directly identified actionable therapeutic targets. In this study, we found that FASN, a key enzyme in *de novo* lipogenesis, is upregulated in ACM myocardium.

We investigated the mechanisms behind the increased expression of FASN and found that JUP binds to the FASN promoter and activates FASN transcription, as demonstrated by DNA pull-down and dual-luciferase reporter assays. Further investigations, including protein interaction predictions, Duolink PLA, and ChIP-qPCR, indicated that JUP interacts with SREBP1 and enhances its transcriptional activity, leading to upregulation of FASN. These findings suggest a link between desmosomal dysfunction and increased fatty acid synthesis. Using ^13^C-glucose isotope tracing in Dsg2^mut/mut^ mice, we observed conversion of glucose into fatty acids. Thus, our data suggest that a substantial portion of the lipids that accumulate in ACM hearts originates from *de novo* synthesis within cardiomyocytes.

We next examined the relationship between lipogenesis and myocardial loss. Isotope tracing and RNA-seq revealed that lipid accumulation is accompanied by transcriptional activation of enzymes involved in ceramide synthesis, leading to increased levels of lipotoxic ceramides. In Dsg2-deficient HL-1 cells, we found that ceramide synthase expression is upregulated and that inhibiting ceramide formation reduces serum deprivation-induced apoptosis. Furthermore, pharmacological inhibition of ceramide synthesis with myriocin decreased apoptosis, and this effect was partially reversed by exogenous C16-ceramide, supporting a causal role for ceramides. Because these *in vitro* apoptosis assays were conducted under serum deprivation, they should be interpreted as a stress-amplified *in vitro* model rather than as direct evidence of spontaneous cardiomyocyte death *in vivo*. Collectively, these results suggest that *de novo* lipogenesis and subsequent ceramide production contribute to cardiomyocyte death in ACM.

Integrating these findings, we propose a model in which desmosomal gene mutations promote JUP nuclear translocation, where JUP cooperates with SREBP1 to upregulate FASN. FASN-mediated *de novo* lipogenesis results in lipid accumulation and lipotoxic substance generation, leading to myocardial loss and other pathological features. This model may help explain several clinical and pathological observations. First, although ACM is now recognized as a biventricular disease, the right ventricle often shows more pronounced lipid accumulation. This relative RV predominance may reflect the lower energy demand of the RV myocardium, which could facilitate lipid accumulation and ceramide production, ultimately resulting in myocardial damage. Second, inflammatory infiltration is present in up to 75% of ACM cases. Lipotoxic species, such as triglycerides and ceramides, may play a significant role in promoting the expression of pro-inflammatory genes, contributing to inflammatory cell infiltration and fibrosis. Indeed, our RNA-seq data showed upregulation of fibrosis-related genes in Dsg2^mut/mut^ hearts, and orlistat treatment significantly reduced collagen deposition, suggesting that FASN inhibition attenuates both the fatty and fibrotic components of ACM pathology.

Lastly, we explored the therapeutic potential of the FASN inhibitor orlistat in Dsg2^mut/mut^ mice. Orlistat treatment significantly improved cardiac structure, contractile function, electrophysiology, and survival. These data suggest that FASN inhibition may be a promising therapeutic strategy for ACM.

### Limitations of the study

Several limitations should be acknowledged. First, our study is primarily based on a Dsg2 mutant mouse model and Dsg2-deficient HL-1 cells. Although we observed FASN upregulation in a small number of ACM patients with PKP2 or DSP mutations (Figure 1A and Table S1), direct mechanistic validation in other desmosomal mutation models (e.g., PKP2 or DSP knockout) is needed. Second, the JUP loss-of-function evidence linking JUP to SREBP1 recruitment and FASN activation was obtained in HL-1 cells and has not been demonstrated *in vivo*; furthermore, our study lacks strong wet-experiment evidence (e.g., co-immunoprecipitation) for a direct physical interaction between JUP and SREBP1. We have primarily used Duolink PLA and ChIP-qPCR, which suggest functional proximity and transcriptional cooperation but are not equivalent to direct binding assays. Future investigations are needed to confirm their physical association. Third, in our study, orlistat was administered systemically; thus, we cannot exclude extra-cardiac effects that might contribute to the observed benefits. Fourth, the clinical association between plasma ceramides and MACE is observational and exploratory. Larger prospective studies are needed to evaluate ceramides as potential biomarkers. In addition, our *in vivo* mechanistic experiments were conducted exclusively in male mice; potential sex-specific effects on the JUP/SREBP1/FASN axis were, therefore, not evaluated. Finally, the therapeutic efficacy of FASN inhibitors has not yet been tested in large animal models or in ACM patients. Despite these limitations, our findings uncover a previously unrecognized JUP/SREBP1/FASN axis that links desmosomal dysfunction to myocardial lipotoxicity and injury, and suggest that targeting FASN may offer a new therapeutic strategy for ACM.

## Resource availability

### Lead contact

Further information and requests for resources and reagents should be directed to and will be fulfilled by the lead contact, Jiangping Song (fwsongjiangping@126.com).

### Materials availability

The Dsg2^mut/mut^ mouse line and the sh-Dsg2 HL-1 cell line generated in this study are available from the lead contact upon reasonable request.

### Data and code availability

• Data reported in this paper will be shared by the lead contact upon request.
• This paper does not report original code.
• Any additional information required to reanalyze the data reported in this paper is available from the lead contact upon request.

## Acknowledgments

This work was supported by the 10.13039/501100001809National Natural Science Foundation of China (824B2010), National Natural Science Fund for Distinguished Young Scholars of China (82125004), and Beijing Natural Science Foundation (Z240015).

## Author contributions

S.H. and J.S. conceived and supervised the project. Z.L. and H.C. designed the study and wrote the manuscript. S.S., J. Han, M.S., and X.C. collected the specimens. Z.L., T.Y., Z.C., and J. Huang performed the pathological and molecular biology experiments. H.C. assisted in data analysis.

## Declaration of interests

The authors declare no competing interests.

## Declaration of generative AI and AI-assisted technologies in the writing process

The authors used DeepSeek for English polishing and proofreading. All output was subsequently reviewed, edited, and approved by the authors, who assume full responsibility for the final content of the manuscript.

## STAR★Methods

### Key resources table

**Table:** 

REAGENT or RESOURCE	SOURCE	IDENTIFIER
**Antibodies**		

Anti-FASN	Proteintech	Cat# 10624-2-AP: RRID: AB_2100801
Anti-JUP (WB/DNA pull-down/ChIP)	Invitrogen	Cat# 13-8500: RRID: AB_2533040
Anti-JUP (PLA)	MCE	Cat# HY-P80683; RRID: AB_3102953
Anti-SREBP1	Abcam	Cat# ab313881
Anti-α-actinin	Abcam	Cat# ab9465; RRID: AB_307264
Anti-Histone H3	Abcam	Cat# ab1791; RRID: AB_302613
Anti-GAPDH	Abcam	Cat# ab9485; RRID: AB_307275
Anti-cleaved caspase-3	Proteintech	Cat# 25128-1-AP; RRID: AB_3073913
Goat anti-Rabbit IgG (H + L), Alexa Fluor 488	Invitrogen	Cat# A-11008; RRID: AB_143165
Goat anti-Mouse IgG (H + L), Alexa Fluor 647	Invitrogen	Cat# A-21235; RRID: AB_2535804
HRP-conjugated goat anti-rabbit IgG (WB)	Abcam	Cat# ab6721; RRID: AB_955447
HRP-conjugated goat anti-mouse IgG (WB)	Abcam	Cat# ab6789; RRID: AB_955439
Rabbit IgG (ChIP control)	Abcam	Cat# ab172730; RRID: AB_2687931

**Chemicals, peptides, and recombinant proteins**		

Orlistat	MCE	Cat# HY-B0218
Myriocin	MCE	Cat# HY-N6798
C16-ceramide	Avanti	Cat# 860516P
^13^C-glucose (U-13C6, 99 atom%)	Sigma-Aldrich	Cat# 389374
Claycomb medium	Sigma-Aldrich	Cat# 51800C
Norepinephrine	Sigma-Aldrich	Cat# A7257
Fibronectin/gelatin coating	Sigma-Aldrich	Cat# FG001
Puromycin	Sigma-Aldrich	Cat# P9620
TRIzol reagent	Invitrogen	Cat# 15596026
RIPA lysis buffer	Beyotime	Cat# P0013B
Lipofectamine 2000	Invitrogen	Cat# 11668019
Lipofectamine RNAiMAX	Invitrogen	Cat# 13778075
RH237	Invitrogen	Cat# S1109

**Critical commercial assays**		

Duolink *In Situ* Red Starter Kit Mouse/Rabbit	Sigma-Aldrich	Cat# DUO92101
ChIP Assay Kit	Cell Signaling Technology	Cat# 9003
*In situ* Cell Apoptosis Detection Kit I (TUNEL)	Boster	Cat# MK1014
Annexin V-FITC Apoptosis Detection Kit	BD Biosciences	Cat# 556547
Dual-Luciferase Reporter Assay System	Promega	Cat# E1910
Nuclear and Cytoplasmic Extraction Reagents Kit	Thermo Fisher	Cat# 78835
PrimeScript RT reagent Kit	Takara	Cat# RR037A
QIAseq Stranded RNA Library Kit	Qiagen	Cat# 180450
BCA Protein Assay Kit	Beyotime	Cat# P0012
Mycoplasma Rapid Detection Kit (PCR)	Procell	Cat# PB180525

**Experimental models: Cell lines**		

HL-1 mouse atrial cardiomyocytes	Sigma-Aldrich	Cat# SCC065
HL-1 sh-Dsg2 (stable Dsg2 knockdown)	This paper	N/A
HL-1 sh-NC (non-targeting control)	This paper	N/A

**Experimental models: Organisms/strains**		

Mouse: cardiomyocyte-specific Dsg2 p.F536C conditional knock-in (Dsg2mut/mut; conditional KI × Myh6-Cre), C57BL/6J	Cyagen Biosciences	N/A (Custom)

**Oligonucleotides**		

qPCR primer: Sptlc2 (mouse)	Beijing Tianyi Huiyuan Biotechnology	F: CCCGTGTCACTGCTTTTGTG R: CTGGCAAGACGGCTACAGAA
qPCR primer: Sptlc1 (mouse)	Beijing Tianyi Huiyuan Biotechnology	F: GAGTCACCGAGCACTATGGG R: TGAGTCAATGCGATGCCCTT
qPCR primer: Fasn (mouse)	Beijing Tianyi Huiyuan Biotechnology	F: CCTGCTATCATCTGACTTCCTCT R: AGGGTGGTTGTTAGAAAGATCAA
qPCR primer: FASN (human)	Beijing Tianyi Huiyuan Biotechnology	F: CATCGGCTCCACCAAGTC R: GCTATGGAAGTGCAGGTTGG
qPCR primer: Dsg2 (mouse)	Beijing Tianyi Huiyuan Biotechnology	F: TCCCGCAGAGTCAGAGAAG R: AAAGTCCAAGCAGACCACCG
qPCR primer: RPL13A (human)	Beijing Tianyi Huiyuan Biotechnology	F: TACTTCACTGTTTAGCCACGAT R: CGAAGATGGCGGAGGTG
qPCR primer: Rpl13a (mouse)	Beijing Tianyi Huiyuan Biotechnology	F: GCTTCTTCTTCCGATAGTGCATC R: AGCCTACCAGAAAGTTTGCTTAC
ChIP-qPCR primer: FASN promoter −73/−42	Beijing Tianyi Huiyuan Biotechnology	F: CCAATTGGTTTCGATGTGG R: GGCGTGGCCTGCTCCACA
shRNA: Dsg2 (mouse)	Beijing Tianyi Huiyuan Biotechnology	5′-TCCGCAGATCAGAGAAGAA-3′
siRNA: JUP (siJUP)	Dharmacon	ON-TARGETplus SMARTpool; Cat# L-040316-01-0020
siRNA: SPTLC1 (siSPTLC1)	Dharmacon	ON-TARGETplus SMARTpool; Cat# L-057477-01-0020
siRNA: SPTLC2 (siSPTLC2)	Dharmacon	ON-TARGETplus SMARTpool; Cat# L-059113-01-0020
siRNA: non-targeting control (siNC)	Dharmacon	ON-TARGETplus Non-targeting Pool; Cat# D-001810-10-05

**Software and algorithms**		

GraphPad Prism	GraphPad	v9.5.0; https://www.graphpad.com/
Proteome Discoverer (with SEQUEST search engine)	Thermo Fisher Scientific	v1.4
STAR	Dobin et al.^16^	v2.7; https://github.com/alexdobin/STAR/
RSEM	Li & Dewey^17^	v1.3.1; https://github.com/deweylab/RSEM
DESeq2	Love et al.^18^	v1.50.2; https://github.com/thelovelab/DESeq2
clusterProfiler	Yu et al.^19^	v4.4; https://github.com/YuLab-SMU/clusterProfiler
ImageJ	NIH	v1.54g; https://imagej.net
FlowJo	BD Biosciences	v10
MATLAB Rhythm	MathWorks	v2.0

### Experimental model and study participant details

#### Human heart tissue acquisition

Heart specimens were procured from the left and right ventricles of patients diagnosed with arrhythmogenic cardiomyopathy (ACM) and patients with non-ACM dilated cardiomyopathy (DCM), alongside non-diseased donor hearts (NC) that were excluded from transplantation due to size mismatch or coronary artery stenosis but free of myocardial ischemia or cardiac dysfunction. Epicardial adipose tissue was carefully separated from myocardial specimens before snap-freezing in liquid nitrogen. The study was approved by the Institutional Review Board of Fuwai Hospital, and written informed consent was obtained from each patient or donor family prior to heart transplantation or donor heart procurement. For omics profiling, ACM and non-diseased donor (NC) ventricular tissues were compared by TMT proteomics (*n* = 4 per group), RNA sequencing (*n* = 4 per group), and lipidomics (*n* = 5 per group). Histological and immunostaining validation used a set of ACM and NC hearts (*n* = 6 per group). Group assignment followed the clinical and pathological diagnosis established before sample analysis; no samples were reassigned after data acquisition. All human participants were of Han Chinese ancestry; race and ethnicity were not otherwise recorded. Both male and female participants were included. Detailed clinical characteristics are provided in Tables S1 and S2.

#### Blood sample collection

Venous blood was collected from ACM probands and their relatives (>14 years old) during clinical consultations and familial screenings between October 2015 and January 2017. Healthy volunteers (>14 years old) without diabetes, obesity, or any cardiovascular disease were recruited at Fuwai Hospital. All participants provided written informed consent. Blood was drawn after at least 2 h postprandial and during physical inactivity, collected into EDTA tubes, centrifuged at 4000 rpm for 10 min to isolate plasma, and stored at −80°C. The plasma cohort comprised ACM patients with MACE (*n* = 21), ACM patients without MACE (*n* = 16), and healthy relatives (*n* = 20; Table S3). Patients were stratified into the MACE and non-MACE groups according to whether a major adverse cardiovascular event (defined as cardiac death, sustained VT, survived sudden cardiac death, syncope, appropriate ICD therapy or HTx from one year before to 6 months after the blood sample collections) had occurred during clinical follow-up prior to lipidomic analysis. Sex and age distributions were comparable across groups (Table S3). All plasma-cohort participants were of Han Chinese ancestry; race and ethnicity were not otherwise recorded. Sex was included as a covariate in the exploratory univariable analysis of MACE and was not significantly associated with the outcome (Figure S6B).

#### Dsg2^mut/mut^ mice

A conditional knock-in mouse line carrying the mouse Dsg2 p.F536C point mutation (corresponding to the human DSG2 p.F531C mutation identified in an ACM family) was generated on a C57BL/6 background by targeted embryonic stem (ES) cell gene targeting (TurboKnockout, Cyagen Biosciences). The point mutation (codon TTC→TGC) was introduced into the endogenous Dsg2 locus together with *loxP* and lox2272 sites, such that the mutant allele is activated only upon Cre-mediated recombination; the *Neo* selection cassette was self-deleted in germ cells, yielding *Neo* cassette–free offspring. Heterozygous mice were backcrossed to C57BL/6J for at least four generations, and homozygous conditional knock-in mice were obtained by intercrossing heterozygotes. To restrict expression of the mutation to cardiomyocytes, homozygous conditional knock-in mice were crossed with the cardiomyocyte-specific Myh6-Cre line. Throughout this study, Dsg2^mut/mut^ denotes homozygous conditional knock-in mice carrying Myh6-Cre, in which the p.F536C mutation is expressed in cardiomyocytes. Age- and sex-matched homozygous conditional knock-in littermates lacking Myh6-Cre, in which the mutation is not activated, were used as WT controls. All mice were housed in a pathogen-free, temperature-controlled facility (22 ± 2°C, 12-h light/dark cycle) with free access to water and standard chow. Only male mice were studied to limit variability from sex hormones and the estrous cycle. Consequently, the influence of sex on the murine phenotype was not assessed, and generalization of these findings to female animals requires further investigation. Unless otherwise specified, cardiac phenotyping (echocardiography, electrophysiology, and histology) was performed on mice at 12 weeks of age. For the orlistat intervention study, treatment began at 6 weeks of age and continued for six weeks. All animal procedures were approved by the Animal Ethics Committee of Fuwai Hospital and conducted in accordance with institutional guidelines.

#### Cell lines

HL-1 mouse atrial cardiomyocytes (obtained from Sigma-Aldrich, catalog #SCC065) were cultured in Claycomb medium (Sigma) supplemented with 10% fetal bovine serum (FBS), 100 U/mL penicillin, 100 μg/mL streptomycin, 0.1 mM norepinephrine, and 2 mM L-glutamine, on 0.02% gelatin/5 μg/mL fibronectin-coated flasks at 37°C in 5% CO_2_. Cells were passaged every 2–3 days at 80–90% confluence using trypsin-EDTA. A stable Dsg2-knockdown HL-1 cell line (sh-Dsg2) was generated by lentiviral transduction with shRNA targeting mouse Dsg2 (sequence: 5′-TCCGCAGATCAGAGAAGAA-3′). A non-targeting shRNA (sh-NC) served as control. Puromycin (2 μg/mL) was used for selection. The parental HL-1 line was obtained directly from Sigma-Aldrich (Cat# SCC065) and used at low passage (fewer than 5 passages after receipt); it was not independently re-authenticated in our laboratory. The sh-Dsg2 and sh-NC derivative lines were verified by confirmation of Dsg2 knockdown (Figures S3F and S3G). All cell lines were tested and confirmed free of mycoplasma contamination using a PCR-based mycoplasma detection assay (Mycoplasma Rapid Detection Kit, Cat# PB180525, Procell, Wuhan, China) prior to experimental use.

### Method details

#### Proteomics profiling

Myocardial tissues (50 mg) were lysed in urea buffer, reduced with DTT, alkylated with iodoacetamide, and digested with trypsin (Promega) overnight at 37°C. Peptides from the 4 NC hearts were pooled as an internal reference (200 μg total). Samples were labeled with TMT reagents (Thermo Fisher) for 1 h, quenched with 5% hydroxylamine, mixed, and separated by UPLC 3000 (Thermo Fisher) followed by LC-MS/MS. MS/MS spectra were searched with SEQUEST in Proteome Discoverer 1.4 against the UniProt human database. Protein expression was calculated relative to the internal reference.

#### Cardiac tissue lipidomics

Frozen tissue (10 mg) was homogenized in chloroform:methanol:water (3:6:1, v/v/v), incubated at 4°C for 30 min, and phase-separated by adding water and chloroform. The organic phase was collected, dried in a SpeedVac, and stored at −80°C. Lipids were reconstituted and analyzed by LC-MS/MS. Total protein from the pellet was quantified by BCA assay for normalization.

#### Plasma lipidomics

Internal standards (Sph 17:1, d7-Cer d18:1/24:0, d7-Cer d18:1/15:0) were added to plasma, followed by extraction with ethyl acetate:isopropanol (2:8, v/v). After centrifugation, the supernatant was analyzed on a Jasper HPLC coupled to a Sciex 4500 MD mass spectrometer in ESI+ mode using a Waters ACQUITY UPLC BEH C18 column (2.1 × 100 mm, 1.7 μm). Ion source settings: curtain gas 20, ion spray voltage 4500 V, temperature 450°C, gas 1 and 2 at 80 and 70, respectively. Individual ceramides were quantitated against spiked internal standards.

#### Western blotting

Tissues or cells were lysed in RIPA buffer (Beyotime #P0013B) with protease and phosphatase inhibitors (Roche). Lysates were resolved by SDS-PAGE (8–15% gels, Beyotime) and transferred to PVDF membranes (0.22–0.45 μm, Millipore). Membranes were blocked in 5% skim milk/TBST for 1 h at 25°C, incubated with primary antibodies overnight at 4°C, then with HRP-conjugated secondary antibodies for 1 h at 25°C. Signal was detected using Western HRP substrate (Millipore) and a Tanon imaging system. Antibodies were listed in key resources table.

#### Immunofluorescence and multiple immunostaining

Paraffin-embedded sections (4 μm) were deparaffinized, rehydrated, and subjected to antigen retrieval in 1 mM EDTA (pH 9.0) using a pressure cooker. After blocking (5% BSA, 15% goat serum in PBS), sections were incubated with primary antibodies overnight at 4°C. Secondary antibodies were Alexa Fluor-conjugated (Invitrogen). Nuclei were stained with DAPI. Images were acquired using a confocal microscope (Leica Stellaris). Fluorescence intensity was quantified using ImageJ by measuring mean fluorescence intensity in α-actinin-positive areas and subtracting background. Antibodies were listed in key resources table.

#### Oil Red O staining

Frozen sections were warmed to room temperature, stained with Oil Red O working solution (3:2 mixture of stock and diluent) for 10–20 min, rinsed with staining wash, and counterstained with hematoxylin. Images were captured with a light microscope. Quantification was performed by measuring Oil Red O-positive area (threshold-based) as a percentage of total tissue area using ImageJ (*n* = 6 per group).

#### Transmission electron microscopy

Tissue specimens were fixed in 2.5% glutaraldehyde/0.1 M sodium cacodylate buffer at 4°C, post-fixed, embedded, and sectioned. Ultrastructure were examined with a JEM-1400 electron microscope (JEOL).

#### TUNEL assay

Apoptotic cells in heart sections were detected using the *In Situ* Cell Apoptosis Detection Kit I (Boster) following the manufacturer’s protocol. TUNEL-positive nuclei (green) were counted per field and normalized to total DAPI-positive nuclei. At least 6 randomly selected fields per section were analyzed (*n* = 6 per group).

#### RNA isolation and qPCR

Total RNA was extracted from tissues or cells using TRIzol (Invitrogen). cDNA was synthesized with Primescript RT-PCR Kit (Takara). qPCR was performed using SYBR Green (Applied Biosystems) on a 7500HT system. Rpl13a was used as internal control. Primer sequences are listed in key resources table.

#### DNA pull-down assay

Biotinylated double-stranded DNA probes covering the −2000 bp region upstream of the FASN transcription start site were synthesized. Nuclear extracts from WT and Dsg2^mut/mut^ mouse hearts were incubated with the probes and streptavidin-magnetic beads. Bound proteins were eluted and identified by LC-MS/MS. For validation, eluted proteins were analyzed by Western blot with anti-JUP antibody.

#### Nuclear-cytoplasmic fractionation

Nuclear and cytoplasmic fractions were isolated using the Nuclear and Cytoplasmic Extraction Reagents Kit (Thermo Fisher). Purity was verified by Western blot for GAPDH (cytoplasmic) and Histone H3 (nuclear). Quantification of JUP in each fraction was performed by densitometry normalized to the respective marker.

#### Dual-luciferase reporter assay

HL-1 cells were seeded in 24-well plates and co-transfected (Lipofectamine 2000) with a firefly luciferase reporter containing either the FASN promoter or a basic control, a Renilla luciferase control, and either JUP expression plasmid or empty vector. After 24 h, luciferase activities were measured with the Dual-Luciferase Reporter Assay System (Promega). Firefly activity was normalized to Renilla, and results expressed as relative luciferase activity.

#### Duolink proximity ligation assay (PLA)

Myocardial sections (4 μm) were fixed with 4% PFA, blocked, and incubated with anti-JUP (MCE HY-P80683, 1:300) and anti-SREBP1 (Abcam ab313881, 1:200). PLA was performed using Duolink *In Situ* Red Starter Kit Mouse/Rabbit (Sigma) according to the manufacturer’s protocol. Images were acquired by fluorescence microscopy (Zeiss LSM880) at 40× magnification, and PLA signals per 50 nuclei were counted using ImageJ.

#### ChIP-qPCR

Chromatin immunoprecipitation was performed using the ChIP Assay Kit (Cell Signaling #9003). Briefly, cells or tissues were crosslinked with 1% formaldehyde, sonicated to 200–700 bp fragments, and immunoprecipitated with anti-JUP (Invitrogen 13–8500) or anti-SREBP1 (Abcam ab313881) or control IgG. Purified DNA was subjected to qPCR with primers spanning the SREBP1 binding motif (−73 to −42 bp) of the FASN promoter (see key resources table). Data are expressed as % input.

#### siRNA and shRNA

For JUP knockdown, sh-Dsg2 HL-1 cells were transfected with 50 nM siRNA targeting mouse JUP (siJUP, Dharmacon ON-TARGETplus smart pool) or control siRNA (siNC) using Lipofectamine RNAiMAX. Knockdown efficiency was evaluated after 48 h by Western blot. For SPTLC1/2 knockdown, cells were transfected with siSPTLC1 and siSPTLC2 (each 25 nM) or siNC. Knockdown was confirmed by qPCR.

#### Myriocin and ceramide rescue experiments

sh-Dsg2 HL-1 cells were pretreated with myriocin (10 μM) for 48 h, followed by serum deprivation for 24 h in the presence or absence of myriocin. For rescue, C16-ceramide (10 μM) complexed with 0.5% BSA was added together with serum deprivation. Apoptosis was measured by Annexin V/PI flow cytometry. Ceramide levels were quantified by LC-MS/MS.

#### Flow cytometry

Cells were harvested, washed with PBS, and stained with Annexin V-FITC and propidium iodide (PI) using the Annexin V-FITC Apoptosis Detection Kit (BD Biosciences) following the manufacturer’s instructions. Samples were analyzed on a FACSCanto II flow cytometer (BD). Apoptotic cells were defined as Annexin V-positive (early and late) and PI-negative (early) or PI-positive (late). Data were processed with FlowJo v10.

#### RNA-seq

Total RNA was isolated from hearts of WT and Dsg2^mut/mut^ mice (*n* = 4 each). Libraries were prepared with the QIASeq Stranded RNA Kit and sequenced on an Illumina NovaSeq 6000 (paired-end, 150 bp). Raw reads were trimmed and aligned to the GRCm38/mm10 genome using STAR (v2.7). Gene expression was quantified with RSEM (v1.3.1). Differential expression was analyzed with DESeq2 (FDR <0.05, |log2FC| ≥ 0.585). GO enrichment analysis was performed using clusterProfiler (v4.4) with an FDR cutoff of 0.05.

#### *In vivo* isotope tracing (^13^C-glucose)

Three days before infusion, a catheter was implanted into the right jugular vein of WT and Dsg2^mut/mut^ mice. After 12 h fasting, ^13^C-glucose (uniformly labeled, 99 atom%) was infused at 0.1 μL min^−1^·g^−1^ for 3 h. Hearts were collected, snap-frozen, and ground. Metabolites were extracted in methanol:acetonitrile:water (40:40:20, 0.5% formic acid), neutralized with ammonium bicarbonate, and analyzed by LC-MS on a Q-Exactive Orbitrap using HILIC separation. Labeled and unlabeled pyruvate, acyl-CoA, malonyl-CoA, and ceramide were quantified.

#### Echocardiography and optical mapping

Transthoracic echocardiography was performed on conscious mice using a Vevo 2100 system (VisualSonics). LVEF, FS, and RV area were measured. For *ex vivo* optical mapping, hearts were excised, perfused retrogradely on a Langendorff apparatus with Tyrode’s solution, stained with voltage-sensitive dye RH237 (0.05 mg/50 mL), and imaged with a MiCAM05 Ultima-L CMOS camera. Pacing was performed at 100 ms and 160 ms cycle lengths. Conduction velocity and APD80/50/20 were analyzed with MATLAB Rhythm 2.0 software.

#### Histology and fibrosis quantification

Hearts were fixed in 4% PFA, embedded in paraffin, and sectioned (4 μm). H&E and Masson’s trichrome staining were performed by standard protocols. Fibrosis area (blue in Masson) was quantified using ImageJ as percentage of total section area. Inflammatory infiltration area was quantified on H&E-stained sections.

### Quantification and statistical analysis

All data are presented as mean ± SEM. Statistical analyses were performed using GraphPad Prism 9.5.0. For two-group comparisons, unpaired two-tailed Student’s *t* test was used (Welch’s correction applied if variances unequal). For multiple groups, one-way or two-way ANOVA was used, followed by post-hoc Tukey’s or Holm-Šídák test as indicated in figure legends. A *p* value <0.05 was considered statistically significant. Exact *p* values are shown in figures with asterisks (∗*p* < 0.05, ∗∗*p* < 0.01, ∗∗∗*p* < 0.001, ∗∗∗∗*p* < 0.0001; ns, not significant). The number of biological replicates (n) is stipulated in each figure legend; technical replicates are noted where applicable. No statistical methods were used to predetermine sample size, but sample sizes were chosen based on previous experience in the field. Randomization and blinding were applied where feasible.
