# Supplementary material for: FASN-mediated de novo lipogenesis promotes ceramide accumulation and myocardial damage in arrhythmogenic cardiomyopathy
Source: iScience. 2026 Aug 28;29(9):117251. doi: 10.1016/j.isci.2026.117251 (PMC13545394; doi:10.1016/j.isci.2026.117251)
Supplement: Supplementary file 1 [file mmc1.pdf]

**iScience, Volume *29***

## Supplemental information

# FASN-mediated *de novo* lipogenesis promotes ceramide accumulation and myocardial damage in arrhythmogenic cardiomyopathy

**Zirui Liu, Hao Cui, Tianshuo Yang, Songren Shu, Zhice Chen, Jing Han, Mingming Su, Jian Huang, Xiao Chen, Jiangping Song, and Shengshou Hu**

# Supplementary Materials

Supplementary Figures

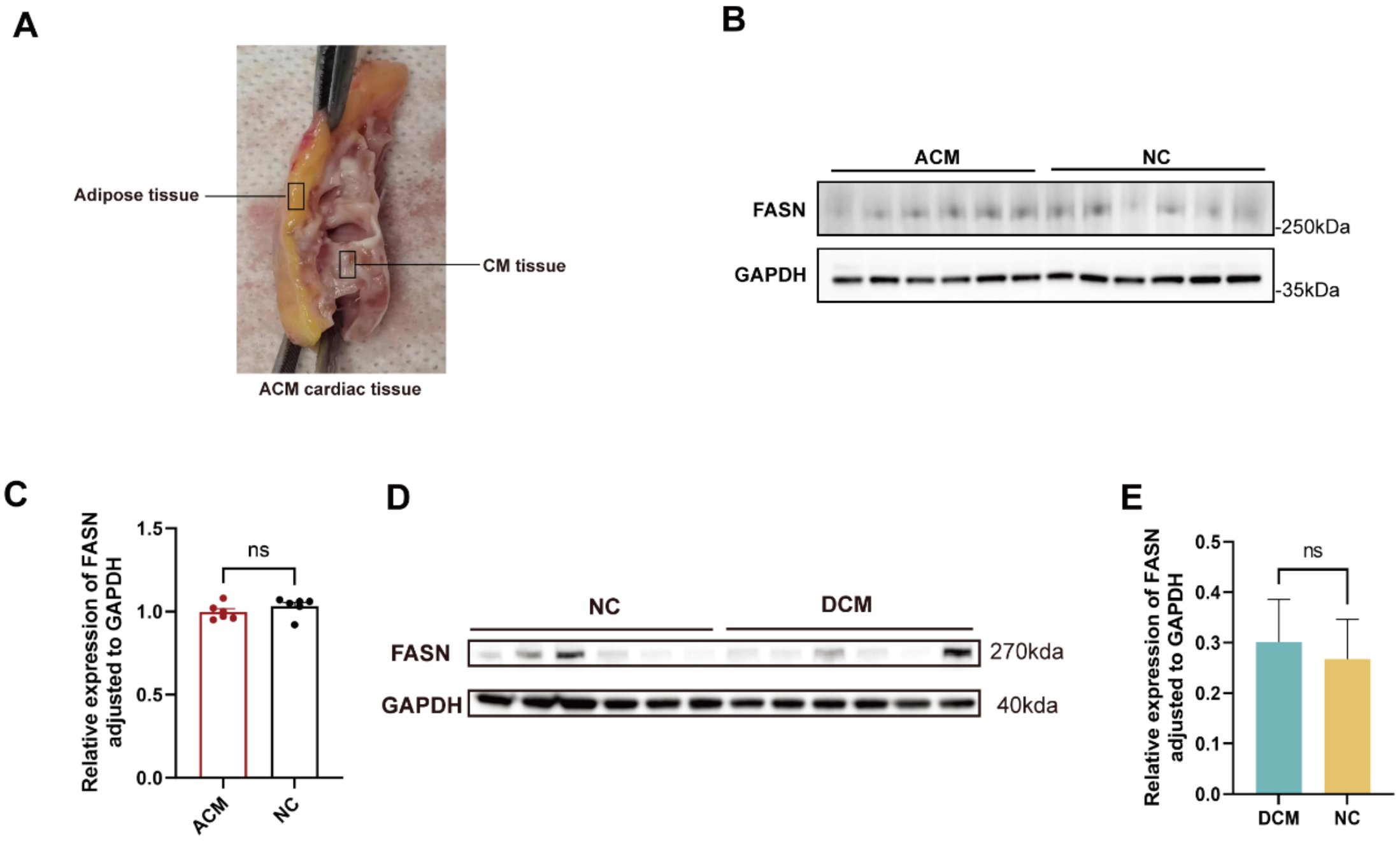


**Figure S1. FASN is specifically upregulated in ACM myocardium, not in adipose tissue or DCM hearts**

(A) Schematic diagram of tissue dissection showing separation of epicardial adipose tissue (Fat) from myocardium (CM) in human heart specimens.

(B) Representative Western blots of FASN and GAPDH in adipose tissue from ACM patients and non-diseased controls (NC, n = 6 per group).

(C) Densitometric quantification of FASN normalized to GAPDH from (B). ns, not significant (Student's t-test).

(D) Representative Western blots of FASN and GAPDH in left ventricular myocardium from dilated cardiomyopathy (DCM) patients and NC (n = 6 per group).

(E) Densitometric quantification of FASN normalized to GAPDH from (D). ns, not significant (Student's t-test).

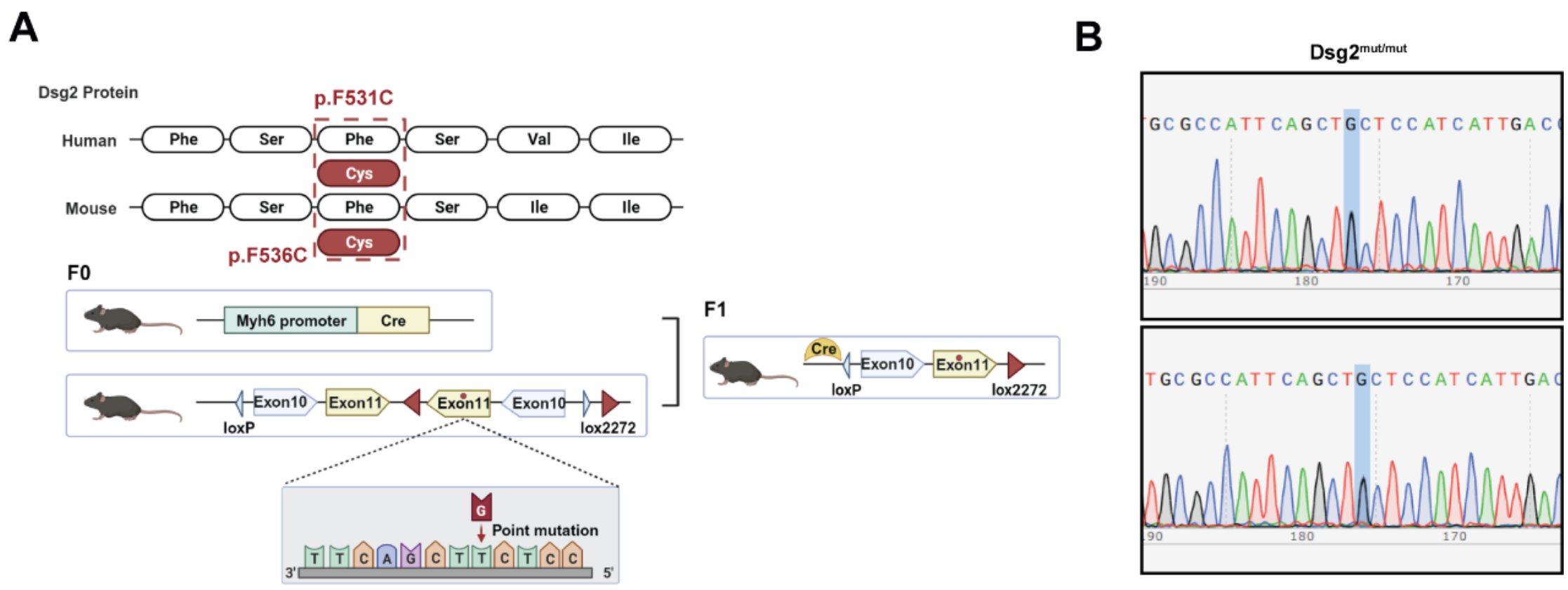


**Figure S2. Generation of Dsg2$^{mut/mut}$ knock-in mice**

(A) Schematic illustration of the targeting strategy for introducing the Dsg2 p.F536C point mutation via the Cre-loxP system. The Neo cassette was self-deleted in germ cells.

(B) Sanger sequencing chromatogram confirming the homozygous mutation (T→G transversion) in Dsg2$^{mut/mut}$ mice.

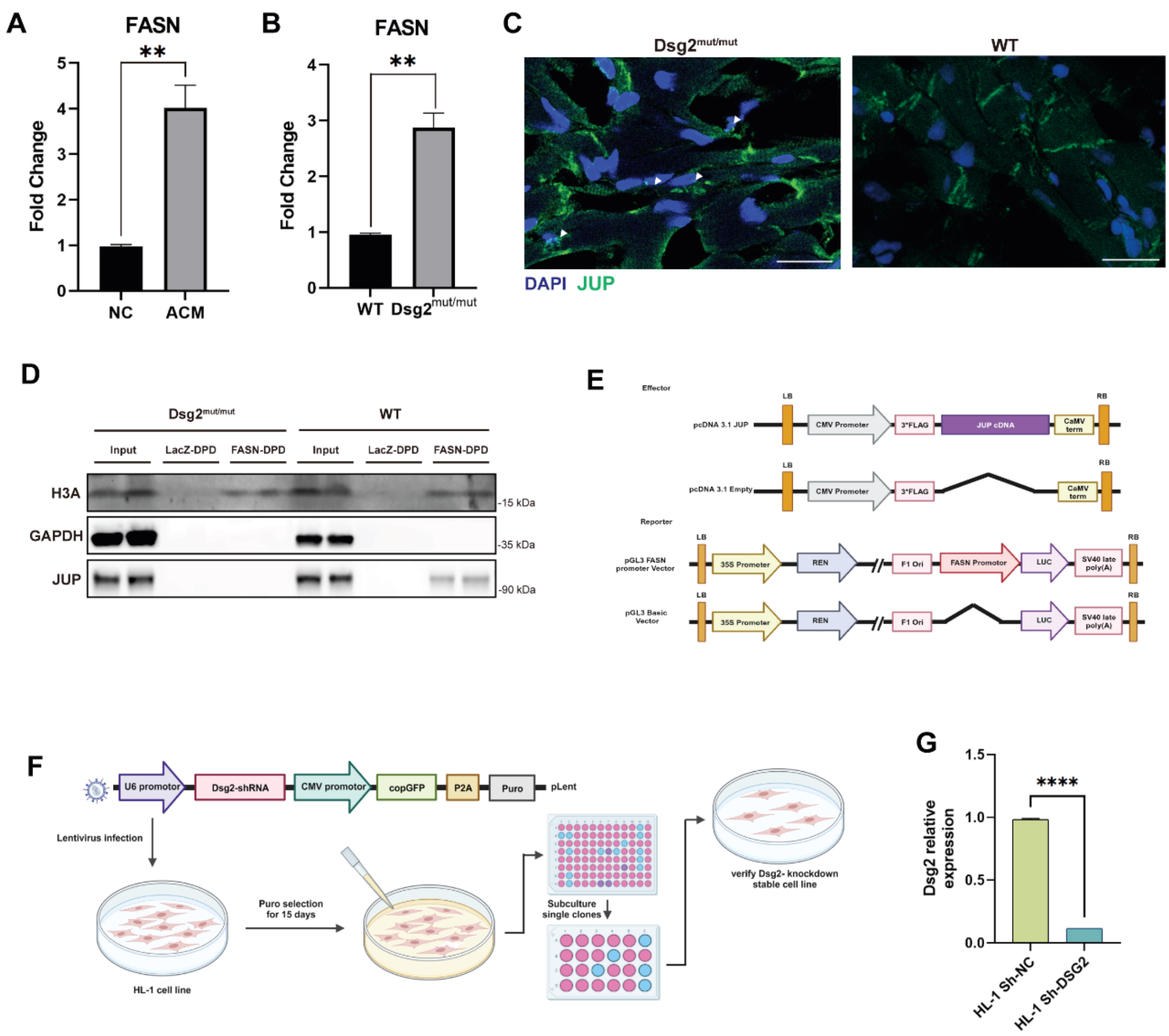


**Figure S3. Additional validation of FASN regulation, JUP binding, and HL-1 sh-Dsg2 cell line**

(A) qPCR analysis of FASN mRNA expression in human ACM and NC myocardium (n = 6 per group). **p < 0.01 (Student's t-test).

(B) qPCR analysis of Fasn mRNA in Dsg2$^{mut/mut}$ and wild-type (WT) mouse hearts (n = 6 per group). ***p < 0.001 (Student's t-test).

(C) Immunofluorescence staining of heart sections from Dsg2$^{mut/mut}$ and WT mice showing JUP (green) and DAPI (blue). Increased nuclear JUP signal is observed in the mutant. Scale bar, 50 μm.

(D) Western blot validation of DNA pull-down using anti-JUP antibody. Increased JUP binding to the FASN promoter probe compared to LacZ control in Dsg2$^{mut/mut}$ heart extracts.

(E) Schematic diagram of the dual-luciferase reporter assay design (corresponds to Figure 3F).

(F) Schematic representation of lentiviral shRNA-mediated Dsg2 knockdown in HL-1 cells.

(G) qPCR validation of Dsg2 knockdown efficiency in HL-1 sh-Dsg2 cells compared to sh-NC control ($n = 3$). ****$p < 0.0001$ (Student's t-test).

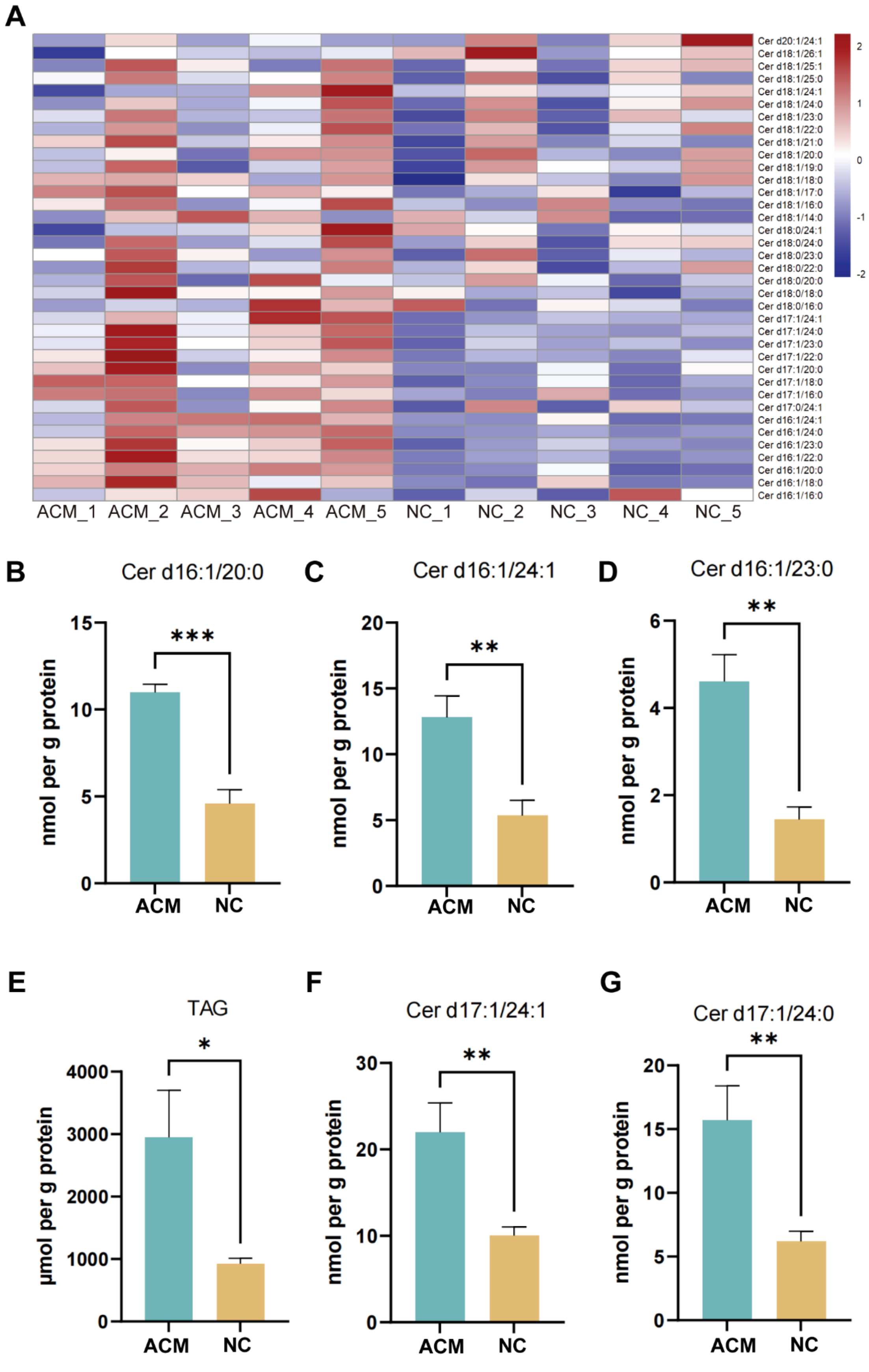


**Figure S4. Detailed lipidomic analysis of human ACM myocardium**

(A) Heatmap showing the distribution of ceramide-related lipid species in myocardial tissues from ACM (n = 5) and NC (n = 5) individuals. Data are $\log_2$-transformed and Z-scored.

(B–G) Absolute quantification of specific lipid species: triacylglycerol (TAG, B), Cer d16:1/20:0 (C), Cer d16:1/23:0 (D), Cer d16:1/24:1 (E), Cer d17:1/24:0 (F), and Cer d17:1/24:1 (G) in ACM vs. NC myocardium. Data are mean ± SEM; *p < 0.05, **p < 0.01, ***p < 0.001, ****p < 0.0001 (Student's t-test). ns, not significant.

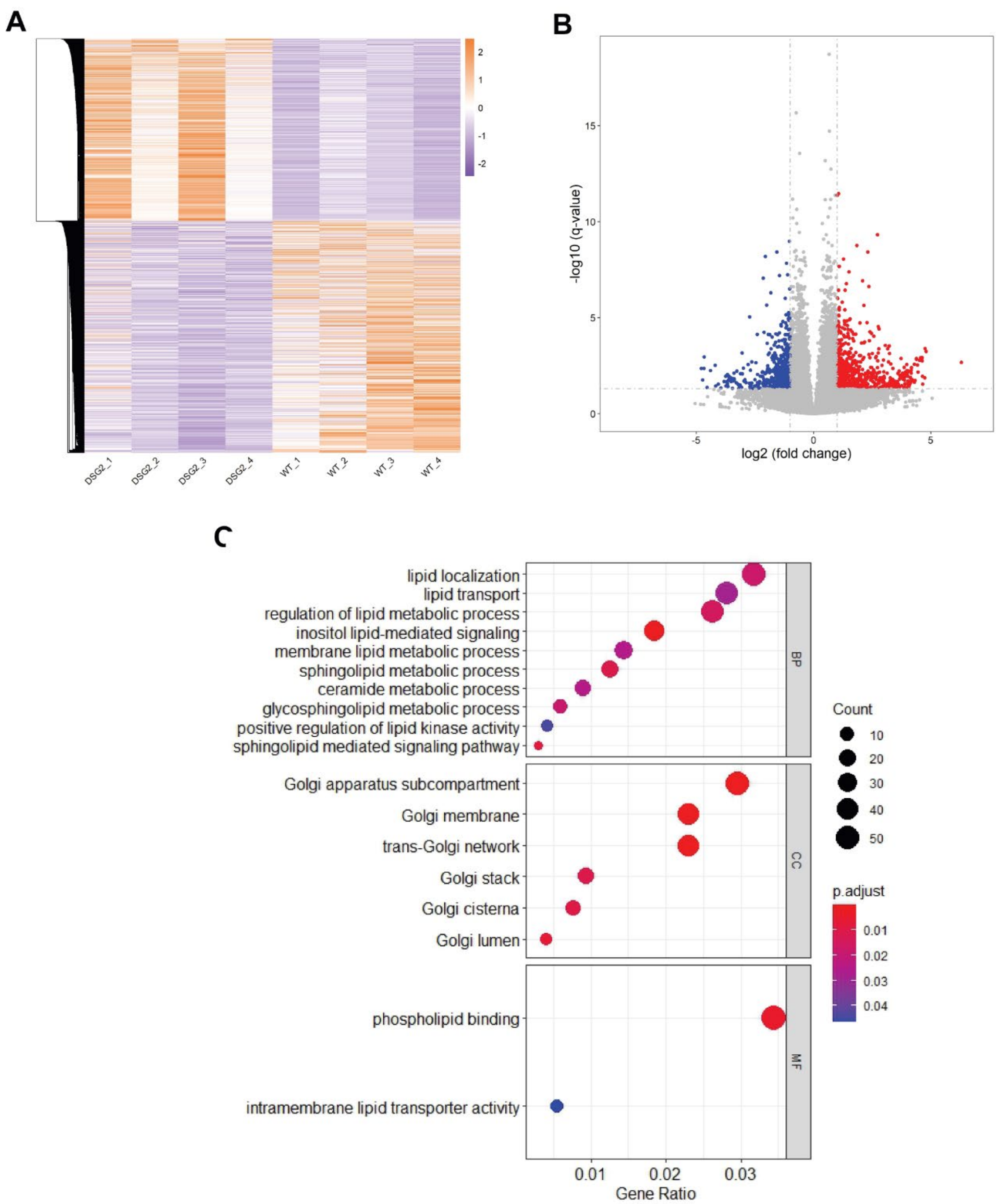

**Figure S5. Global gene expression changes in Dsg2$^{mut/mut}$ mice hearts.**

(A) Unsupervised hierarchical clustering and heatmap analysis of cardiac mRNA expression profiles (4 vs. 4 samples).

(B) Volcano plot displaying the log2(fold change) vs. -log10(q-value). Differentially expressed genes were filtered using standard thresholds.

(C) GO enrichment analysis reveals activated biological pathways and molecular functions in Dsg2$^{mut/mut}$ mice hearts.

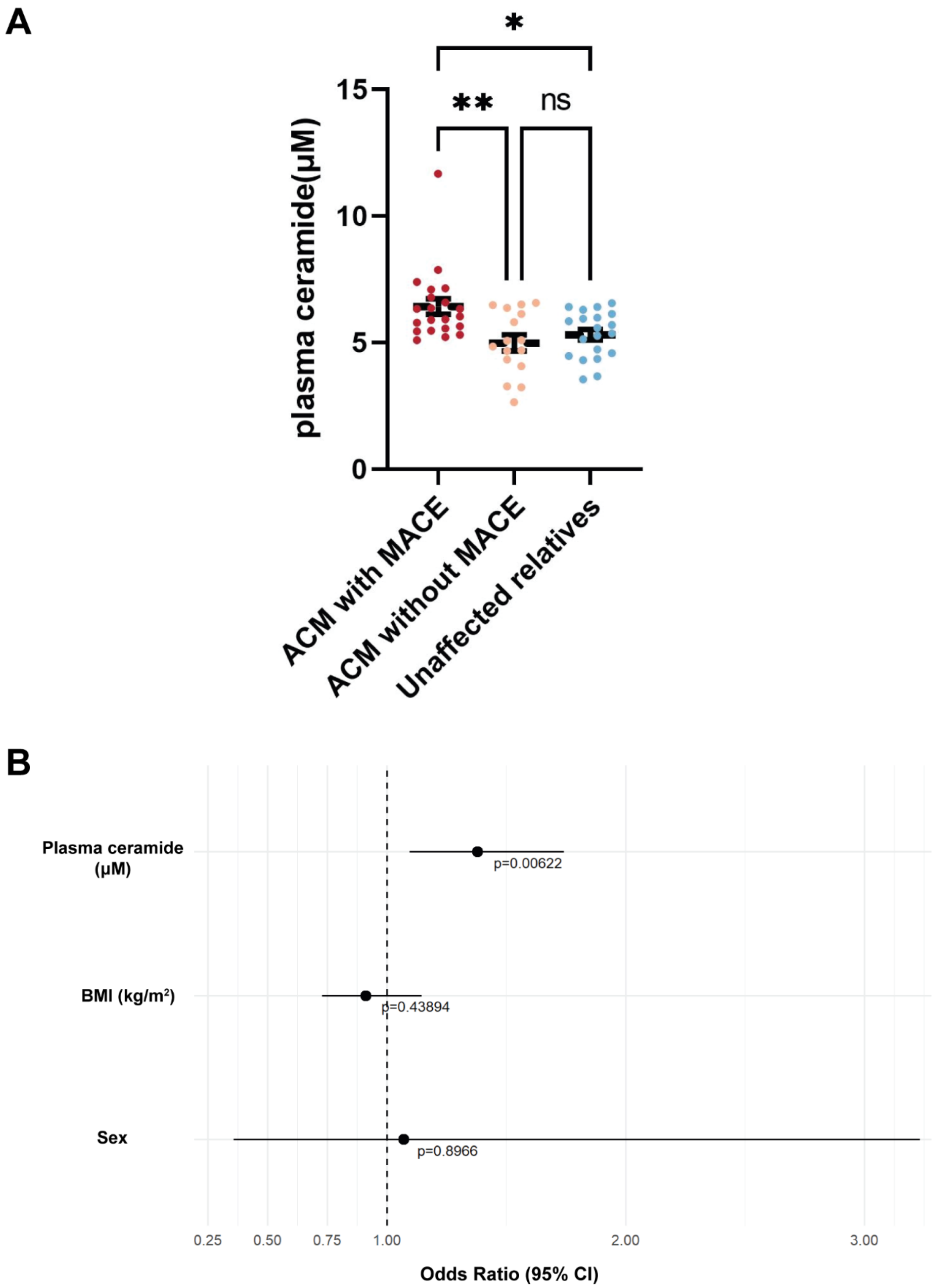


**Figure S6. Clinical significance of plasma ceramides in risk stratification for MACE.**

(A) Comparative analysis of plasma ceramide concentrations among ACM patients with MACE, ACM patients without MACE, and unaffected relatives.

(B) Forest plot derived from univariable analysis evaluating the predictive value of plasma ceramides, BMI, and sex for MACE. Data are presented as mean ± SEM. *P < 0.05, **P < 0.01. ACM, arrhythmogenic cardiomyopathy; MACE, major adverse cardiovascular events; BMI, body mass index; NC, normal control.

**Table S1. Clinical diagnosis and characteristics of patients with ACM in this study.**

| Individual | Mutation | MACE* | Family History | Ventricular Structural Alterations | Tissue Characterization | Repolarization Abnormalities | Depolarization/Conduction Abnormalities | Arrhythmia | Diagnosis† |
|---|---|---|---|---|---|---|---|---|---|
| | | | major/minor | major/minor | major/minor | TWI | QRS duration | | major/minor |
| **Proteomics study** | | | | | | | | | |
| ACM-1 | DSG2 | HTx | 1/0 | 1/0 | 1/0 | V1–V3 RBBB | 1 | VE | 3/2 |
| ACM-2 | PKP2 | HTx | 0/0 | 1/0 | 1/0 | V4–V6 | 1 | VE/NSVT | 2/2 |
| ACM-3 | DSG2 | HTx | 0/0 | 1/0 | 1/0 | V4–V6 | 1 | VE/NSVT | 2/3 |
| ACM-4 | PKP2 | HTx | 1/0 | 1/0 | 1/0 | V1–V4 RBBB | 1 | VE/NSVT | 3/3 |
| **Western blot, lipidomics, transcriptomics and immunostaining study of cardiac tissues** | | | | | | | | | |
| ACM-5 | DSG2 | HTx | 0/1 | 1/0 | 1/0 | V4–V6 | 1 | NSVT | 3/3 |
| ACM-6 | DSG2 | HTx | 1/0 | 1/0 | N/A | N/A | N/A | s-VT/VE | 3/0 |
| ACM-7 | DSG2 | HTx | 0/0 | 1/0 | 1/0 | V4–V6 | 1 | VE/NSVT | 2/2 |
| ACM-8 | DSG2 | HTx | 0/0 | 1/0 | 1/0 | V4–V6 | 1 | VE/NSVT | 2/3 |
| ACM-9 | DSG2 | HTx | 1/0 | 1/0 | 1/0 | V1–V4 RBBB | 1 | VE/NSVT | 3/3 |
| ACM-10 | DSG2 | HTx | 0/1 | 1/0 | 1/0 | V4–V6 | 1 | NSVT | 3/3 |
| ACM-11 | DSG2 | HTx | 0/0 | 1/0 | 1/0 | V1–V3 RBBB | 1 | NSVT | 3/3 |
| **Plasma lipidomics** | | | | | | | | | |
| ACM-12 | PKP2 | 0 | 1/0 | 0/0 | N/A | 0 | 0 | VE | 1/1 |
| ACM-13 | PKP2 | 0 | 1/0 | 0/1 | N/A | 0 | 0 | NSVT | 2/1 |
| ACM-14 | PKP2 | 0 | 1/0 | 1/0 | N/A | V1–V3 | 1 | NSVT | 3/2 |
| ACM-15 | DSP | 0 | 0/0 | 0/0 | N/A | V1–V3 | 0 | NSVT | 1/1 |
| ACM-16 | PKP2 | 0 | 0/0 | 1/0 | N/A | V1–V3 | 0 | NSVT | 3/0 |
| ACM-17 | DSG2 | 0 | 0/0 | 1/0 | N/A | V1–V4 RBBB | 1 | NSVT | 1/3 |
| ACM-18 | PKP2 | 0 | 1/0 | 1/0 | N/A | V1–V3 | 0 | NSVT/VE | 3/1 |
| ACM-19 | PKP2 | 0 | 0/0 | 1/0 | N/A | V1–V3 | 1 | NSVT/VE | 2/2 |
| ACM-20 | TMEM43 | 0 | 1/0 | 0/1 | N/A | 0 | 0 | VE | 2/1 |
| ACM-21 | DSG2 | 0 | 1/0 | 1/0 | 1/0 | 0 | 0 | VE | 3/1 |
| ACM-22 | PKP2 | 0 | 1/0 | 1/0 | N/A | V2–V3 | 0 | NSVT/VE | 2/2 |
| ACM-23 | DSG2 | 0 | 1/0 | 0/0 | N/A | V1–V4 RBBB | 1 | NSVT/VE | 2/2 |
| ACM-24 | PKP2 | 0 | 1/0 | 0/0 | N/A | 0 | 0 | NSVT/VE | 2/0 |
| ACM-25 | DSG2 | 0 | 1/0 | 1/0 | N/A | V1–V4 RBBB | 0 | NSVT/VE | 3/1 |
| ACM-26 | PKP2 | 0 | 0/0 | 1/0 | N/A | V1–V2 | 0 | NSVT/VE | 2/1 |
| ACM-27 | PKP2 | 0 | 1/0 | 1/0 | N/A | V1–V3 | 1 | NSVT/VE | 3/2 |
| ACM-28 | DSG2 | HTx | 1/0 | 1/0 | 1/0 | N/A | N/A | s-VT/VE | 4/0 |
| ACM-29 | DSG2 | HTx | 1/0 | 1/0 | 1/0 | N/A | N/A | s-VT/VE | 4/0 |

| | | | | | | | | | |
|---|---|---|---|---|---|---|---|---|---|
| ACM-30 | TMEM43 | HTx | 1/0 | 1/0 | 1/0 | N/A | N/A | s-VT/VE | 4/0 |
| ACM-31 | DSG2 | HTx | 1/0 | 1/0 | 1/0 | N/A | N/A | s-VT/VE | 4/0 |
| ACM-32 | DSG2 | HTx | 0/1 | 1/0 | 1/0 | V1–V4 RBBB | 0 | s-VT/VE | 3/1 |
| ACM-33 | DES | HTx | 1/0 | 1/0 | 1/0 | V1–V4 RBBB | 1 | s-VT | 4/2 |
| ACM-34 | DSG2 | HTx | 0/1 | 1/0 | 1/0 | V1–V4 RBBB | 0 | s-VT/VE | 3/1 |
| ACM-35 | PKP2 | HTx | 1/0 | 1/0 | N/A | V1–V3 | 1 | s-VT/VE | 3/2 |
| ACM-36 | PKP2 | ICD | 1/0 | 1/0 | 1/0 | V1–V4 RBBB | 1 | VE/NSVT | 3/3 |
| ACM-37 | DSG2 | ICD | 1/0 | 1/0 | N/A | N/A | N/A | s-VT/VE | 3/0 |
| ACM-38 | PKP2 | ICD | 1/0 | 1/0 | N/A | V2–V3 | 0 | NSVT/VE | 2/2 |
| ACM-39 | DSG2 | HTx | 0/0 | 1/0 | 1/0 | V1–V4 RBBB | 1 | s-VT/VE | 3/2 |
| ACM-40 | PKP2 | ICD | 0/0 | 1/0 | N/A | V1–V4 RBBB | 1 | s-VT/VE | 2/2 |
| ACM-41 | RYR2 | HTx | 1/0 | 1/0 | 1/0 | N/A | N/A | s-VT/VE | 4/0 |
| ACM-42 | DSG2 | HTx | 1/0 | 1/0 | N/A | N/A | N/A | s-VT/VE | 3/0 |
| ACM-43 | DSG2 | ICD | 1/0 | 1/0 | N/A | V1–V4 RBBB | 1 | s-VT | 3/2 |
| ACM-44 | PKP2 | HTx | 0/1 | 1/0 | 1/0 | V4–V6 | 1 | NSVT | 4/2 |
| ACM-45 | DES | HTx | 1/0 | 1/0 | 1/0 | V1–V3 | 0 | NSVT/VE | 4/1 |
| ACM-46 | DSP | ICD | 1/0 | 1/0 | N/A | V2–V3 | 0 | NSVT/VE | 2/2 |
| ACM-47 | PKP2 | ICD | 1/0 | 1/0 | N/A | N/A | N/A | s-VT/VE | 3/0 |
| ACM-48 | DSG2 | HTx | 1/0 | 1/0 | 1/0 | V1–V4 RBBB | 1 | s-VT | 3/2 |

* MACE events were defined as cardiac death, sustained VT, survived sudden cardiac death, syncope, appropriate ICD therapy or HTx from one year before to 6 months after the blood sample collections.

† Definite diagnosis contains 2 major or 1 major plus 2 minor or 4 minor from different categories; borderline diagnosis is defined as 1 major plus 1 minor or 3 minor criteria.

ACM=arrhythmogenic cardiomyopathy; HTx=heart transplant; ICD= implantable cardioverter defibrillator; N/A = no related information available; NSVT=non-sustained ventricular tachycardia; RBBB= right bundle branch block; s-VT= sustained ventricular tachycardia; TWI=T wave inversion; VE=ventricular extrasystoles.

**Table S2. Baseline characteristics of individuals in the cardiac lipidomics study.**

| Variables | ACM (n=5) | Healthy Donors (n=5) | P (ACM vs Healthy Donors) |
|---|---|---|---|
| Male (%) | 3 (60) | 3 (60) | 1 |
| Age (y) | 38.51±3.16 | 40.00±4.52 | 0.791 |
| BMI (kg/m$^2$) | 22.25±0.82 | 22.52±0.85 | 0.823 |
| LVEF (%) | 42.00±4.58 | 62.66±1.73 | <0.001 |

BMI=Body mass index; LVEF=Left ventricular ejection fraction.

**Table S3. Baseline characteristics of individuals included in the comparison of plasma ceramide concentrations in the cohort.**

| Characteristic | ACM with MACE (n=21) | ACM without MACE (n=16) | Healthy Relatives (n=20) | P (ACM with MACE vs ACM without MACE) | P (ACM with MACE vs Healthy Relatives) | P (ACM without MACE vs Healthy Relatives) |
|---|---|---|---|---|---|---|
| ceramide (μM) | 6.42±0.34 | 4.98±0.31 | 5.31±0.27 | **0.002** | 0.014 | 0.7092 |
| Male (%) | 13 (61.90) | 11 (68.75) | 11 (55.00) | 1.00 | 1.00 | 1.00 |
| Age (y) | 31.31±3.29 | 38.54±3.30 | 36.46±2.64 | 0.134 | 0.248 | 0.234 |
| BMI (kg/m$^2$) | 23.44±2.11 | 24.09±2.93 | 22.69±2.32 | 0.441 | 0.512 | 0.218 |
| Syncope (%) | 6 (28.6) | 2 (12.5) | | 1.00 | | |
| ICD placed (%) | 10 (47.6) | 0 (0) | | 1.00 | | |
| LVEF (%) | 45.11±3.47 | 63.5±1.92 | | <0.001 | | |
| LVEDD (mm) | 50.79±2.91 | 45.00±1.66 | | 0.141 | | |
| RVID (mm) | 45.26±4.57 | 32.25±3.64 | | 0.062 | | |
| NT-proBNP (pmol/ml) | 954.6±343.2 | 502.92±130.2 | | 0.318 | | |
| BUN (mmol/L) | 7.12±0.55 | 5.29±0.40 | | 0.019 | | |
| Creatinine (μmol/l) | 91.11±6.58 | 87.94±4.41 | | 0.721 | | |
| ALB (g/L) | 49.62±2.63 | 44.68±1.81 | | 0.171 | | |
| ALT (IU/L) | 29.95±3.69 | 28.15±5.26 | | 0.769 | | |
| AST (IU/L) | 25.32±2.72 | 26.46±5.75 | | 0.841 | | |

ALB=albumin; ALT=Alanine Transaminase; AST=Aspartate Transaminase; BMI=Body mass index; BUN=Blood urea nitrogen; DCM=Dilated cardiomyopathy; ICD=Implantable cardioverter defibrillator; LVEDD=Left ventricular end-diastolic diameter; LVEF=Left ventricular ejection fraction; RVID=Right ventricular internal diameter
